# SSR white paper: guidelines for utilization and performance of direct MR arthrography

**DOI:** 10.1007/s00256-023-04420-6

**Published:** 2023-08-11

**Authors:** Eric Y. Chang, Jenny T. Bencardino, Cristy N. French, Jan Fritz, Chris J. Hanrahan, Zaid Jibri, Ara Kassarjian, Kambiz Motamedi, Michael D. Ringler, Colin D. Strickland, Christin A. Tiegs-Heiden, Richard E.A. Walker

**Affiliations:** 1 Radiology Service, VA San Diego Healthcare System, San Diego, CA USA; 2 Department of Radiology, University of California, San Diego Medical Center, San Diego, CA USA; 3 Department of Radiology, Hospital of the University of Pennsylvania, Philadelphia, PA USA; 4 Department of Radiology, Penn State Hershey Medical Center, Hummelstown, PA USA; 5 Department of Radiology, New York University Grossman School of Medicine, New York, NY USA; 6 Summit Physician Specialists, Murray, UT USA; 7 GNMI in Mississauga, Greater Toronto Area, Toronto, ON Canada; 8 Department of Radiology, Division of Musculoskeletal Imaging, Olympia Medical Center, Elite Sports Imaging, Madrid, Spain; 9 Department of Radiology, University of California, Los Angeles Medical Center, Los Angeles, CA USA; 10 Department of Radiology, Mayo Clinic, Rochester, MN USA; 11 Department of Radiology, University of Colorado School of Medicine, Aurora, CO USA; 12 Department of Radiology, Mayo Clinic, Rochester, MN USA; 13 McCaig Institute for Bone and Joint Health, Calgary, Canada; 14 Cumming School of Medicine, University of Calgary, 3280 Hospital Dr NW, Calgary, AB T2N 4Z6 Canada

**Keywords:** Direct MR arthrography, MRI, Labrum, Post-operative, Ligament, Plica, Meniscectomy

## Abstract

**Objective:**

Direct magnetic resonance arthrography (dMRA) is often considered the most accurate imaging modality for the evaluation of intra-articular structures, but utilization and performance vary widely without consensus. The purpose of this white paper is to develop consensus recommendations on behalf of the Society of Skeletal Radiology (SSR) based on published literature and expert opinion.

**Materials and methods:**

The Standards and Guidelines Committee of the SSR identified guidelines for utilization and performance of dMRA as an important topic for study and invited all SSR members with expertise and interest to volunteer for the white paper panel. This panel was tasked with determining an outline, reviewing the relevant literature, preparing a written document summarizing the issues and controversies, and providing recommendations.

**Results:**

Twelve SSR members with expertise in dMRA formed the ad hoc white paper authorship committee. The published literature on dMRA was reviewed and summarized, focusing on clinical indications, technical considerations, safety, imaging protocols, complications, controversies, and gaps in knowledge. Recommendations for the utilization and performance of dMRA in the shoulder, elbow, wrist, hip, knee, and ankle/foot regions were developed in group consensus.

**Conclusion:**

Although direct MR arthrography has been previously used for a wide variety of clinical indications, the authorship panel recommends more selective application of this minimally invasive procedure. At present, direct MR arthrography remains an important procedure in the armamentarium of the musculoskeletal radiologist and is especially valuable when conventional MRI is indeterminant or results are discrepant with clinical evaluation.

## Introduction

The technique of direct magnetic resonance arthrography (dMRA) was first described in 1987 [1]. Despite the element of invasiveness that was introduced to an otherwise non-invasive imaging modality, several benefits were readily apparent. These included improved delineation of the surfaces of intra-articular structures related to joint distension, improvements in signal-to-noise as well as contrast-to-noise ratios as a result of the T1 shortening effects of dilute gadolinium, and added benefit of indirect arthrographic signs in the absence of direct depiction of the responsible defect (e.g., extra-articular leakage of contrast material and abnormal filling of bursae or adjacent joint compartments). Using a sample of cadaveric shoulders, wrists, knees, and ankles imaged on a 1.5-T system, the authors suggested higher accuracy for the diagnoses of several intra-articular abnormalities compared with conventional magnetic resonance imaging (cMRI) [1]. Clinical indications rapidly expanded over the subsequent years, often in parallel with surgical advancements such as arthroscopy [2–7], and dMRA became a routinely utilized and integral part of most clinical practices. After several decades of widespread use, dMRA is now accepted as a highly accurate imaging modality for the evaluation of intra-articular structures.

The drawbacks of dMRA have remained largely unchanged through the years, mostly related to the minimally invasive arthrogram component. Since the introduction of dMRA, there have been significant advancements in MRI technology, including widespread availability of 3-T scanners and better surface coils, that have vastly improved image quality. As a consequence, the advantages of dMRA over cMRI have evolved leading to variability in its utilization for different clinical indications and in different practices. In recognition of this, the Standards and Guidelines Committee of the Society of Skeletal Radiology (SSR) commissioned the current white paper. Twelve SSR members with expertise in dMRA were selected to form an ad hoc white paper panel and were tasked with reviewing the published literature and provide recommendations on the utilization of dMRA based on consensus expert opinion.

**Fig. 1:**
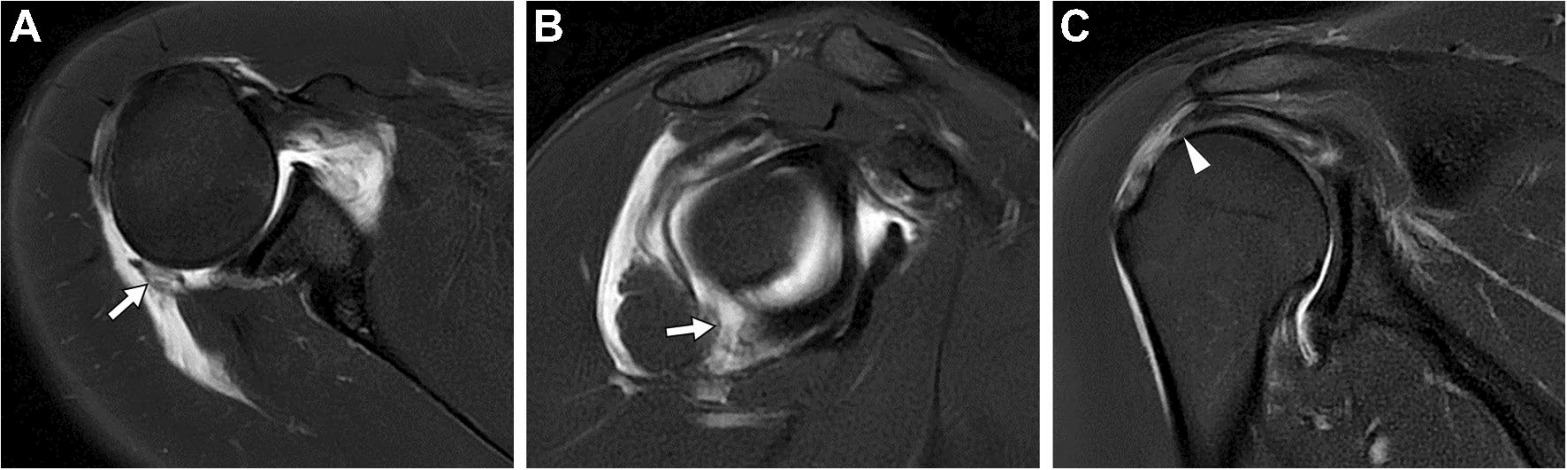
Arthrographic effect from a post-traumatic hemarthrosis/effusion. Twenty-five-year-old man who fell while playing football. Axial (**A**), sagittal (**B**), and coronal (**C**) T2-weighted fat-suppressed conventional 3 T MR images show a tear of the inferior glenohumeral ligament complex involving the posterior band and capsule (arrows) as well as traumatic rupture of the superior cuff (arrowhead)

It should be noted that in the acute setting, a joint effusion may create an arthrographic effect. Some authors have suggested dMRA of the shoulder is unnecessary in this scenario [8, 9] (Fig. 1). This principle can be extrapolated to include all joints. Similarly, in the presence of moderate to severe arthritis, the value of dMRA is decreased, and if magnetic resonance (MR) imaging must be performed, the authorship panel recommends cMRI. A retrospective study in patients 50 years and older referred for hip pain found that 100% of patients with moderate to severe osteoarthritis (Tonnis grade 2–3 or joint space width ≤ 2 mm) demonstrated acetabular labral pathology on dMRA. Since arthroscopic surgical repair is limited in this setting, the authors concluded that dMRA may not be indicated [10]. We stress that panel recommendations are generalized for routine clinical practice and refer specifically to the utilization of cMRI or dMRA as the *initial* type of MR imaging exam. Notably, in the setting of an indeterminate cMRI exam, dMRA may be both an invaluable tool and appropriate for confirming or excluding intra-articular pathology that may alter management. We also emphasize that a practitioner may appropriately choose to supersede a panel recommendation based on their expertise and experience in a specific clinical scenario. Consultation with the referring physician, who may have a strong preference for or against dMRA in a particular patient or clinical scenario, is important.

## Materials and methods

The white paper authorship panel first convened on March 14, 2022, at the Society of Skeletal Radiology Annual Scientific Meeting in San Diego, CA, USA, with an additional 7 virtual or blended in-person/virtual meetings over the following 13 months. The manuscript outline was first delineated, and six subgroups established, each pairing a senior and more junior panel member. Subgroups were assigned section(s), including one major joint (shoulder, elbow, wrist, hip, knee, or ankle/foot), and tasked with completing an exhaustive literature review, preliminary draft, and presentation of evidence to the authorship panel for discussion, deliberation and development of panel recommendations by consensus agreement. In an effort to substantiate panel member support of individual recommendations, an anonymous poll was conducted prior to the final meeting and recommendations that were not unanimous were further deliberated and a final panel recommendation established. Panel recommendations are provided as one of three categories (dMRA recommended, dMRA or cMRI recommended, cMRI recommended), with comments added for clarification as needed. All recommendations were made by consensus. For transparency, the level of agreement is classified as *unanimous* (12/12), *supermajority* (≧ 10/12) or *majority*, in order to reflect dissenting opinions on the consensus recommendation [11].

Subgroups completed their primary MEDLINE/PubMed searches between April 30, 2022, and June 28, 2022, using the terms, “MR” and “arthrography” and the specific joint(s) they were assigned. All abstracts were reviewed. Scientific articles, review articles, and systematic reviews that included MR arthrography and were available online or through the local institution’s interlibrary loan program were obtained. Articles were reviewed and any additional references from these articles that were not originally captured in the initial search were also obtained and reviewed. In addition, limited but dedicated searches were performed to ensure a comprehensive review of available literature. Articles evaluating the utility and/or diagnostic performance of dMRA and those directly comparing cMRI and dMRA were favored, particularly those with surgical confirmation.

The following is a summary of the literature and recommendations from the panel, organized by (1) technical considerations in the performance of dMRA; (2) pathology-specific, joint independent indications; and (3) joint-specific considerations in the shoulder, elbow, wrist, hip, knee, and ankle/foot regions. We conclude by discussing controversies and gaps in the literature. Of note, the focus of this white paper is on dMRA and not the other forms of arthrography, including indirect MRA and CT arthrography.

## Technical considerations

### Image guidance

Image-guidance for the arthrogram component of the exam is recommended for higher accuracy compared to blind injections. Fluoroscopy and ultrasound (US) guidance are the most commonly used modalities [12], but computed tomography (CT) and MR imaging may be useful when no other imaging modalities are available [13–15]. When radiation is used, the “as low as reasonably achievable” (ALARA) principle applies to reduce dose to both the patient and operator. Operators must be properly trained and routinely use all available dose reduction techniques [16, 17]. Ultrasound-guided arthrographic injection eliminates the exposure to ionizing radiation which may be especially pertinent in teenagers and young adults [18], but a drawback is the limited ability to document flow into neighboring compartments. Joint-specific considerations are discussed in the respective sections below.

### General procedure

As with all procedures, an initial assessment for potential contraindications should be performed. The main contraindications for dMRA are suspected peri-articular or joint proper infections, reflex sympathetic dystrophy, severe coagulopathy, and allergic reaction to any of the injected components. Positioning of the patient depends on the approach. In our experience, taking the necessary time to optimize positioning to ensure ease of joint access and patient comfort is critical. After the skin start point has been selected and marked, the area should be sterilized with a cleaning solution and then draped [19]. Local anesthesia may be provided to the skin and underlying subcutaneous tissues using 1% lidocaine, with sodium bicarbonate buffer at the preference of the proceduralist [19]. Twenty to 25-gauge needles are used to access the joint, with the length depending on the particular joint and approach [12, 20]. If present, pre-existing joint fluid could be aspirated to prevent dilution of the injectate. Introduction of gas should be avoided by limiting the number of syringe exchanges, clearing bubbles from any extension tubing, and filling the needle hub with injectate prior to making connections. After the joint is accessed, pure iodinated contrast may be injected in small increments (e.g., 0.2 mL) via extension tubing while obtaining fluoroscopy spot views to ensure proper intra-articular flow. If resistance is met, rotating the needle may aid in penetration through the joint capsule [19]. In the case of an US-guided injection, if fluid accumulates around the tip of the needle, the needle is likely periarticular and needs to be adjusted accordingly. After confirmation of intra-articular needle positioning, a mixture of diluted gadolinium-based contrast agent, normal saline, iodinated contrast, and/or anesthetic may be injected. Too little injected volume will result in inadequate expansion of the joint, whereas too large of a volume will create iatrogenic leakage from the joint space that may be interpreted as a local tear or a lesion [21]. For the appropriate injection volume for each joint, the reader may refer to the respective sections in this article and Table 1. The needle is subsequently removed, and a bandage should be placed over the insertion point. Image acquisition should be performed as soon as possible after the injection to maximize capsular distention and minimize absorption of contrast [22, 23], ideally within 30 min. Clear instructions to the patient regarding remaining still during MRI acquisition is crucial to prevent deleterious motion-related image degradation.

**Table 1:** Recommended injection volumes for the major joints

Joint	Total volume (mL)^1^
Shoulder (glenohumeral)	8–15
Elbow	3–6
Wrist (radiocarpal)	3–4^2^
Hip	10–12
Knee	30–40
Ankle (talocrural)	4–8^3^
Metatarsophalangeal joints	1–2

^1^Ranges provided are for a typical joint, but optimal volumes in a particular patient may differ and additional feedback mechanisms should be employed (e.g., injection ceases at resistance or when fluoroscopy shows complete distension)

^2^If communication with the midcarpal or distal radioulnar joints, add 3 mL or 1 mL, respectively

^3^Volumes towards the lower and mid-range typically used, except when there is communication with adjacent compartments

### Injectate and safety

Most practitioners prefer to include gadolinium-based contrast agents (GBCAs) for dMRA, but some use a saline-only technique [24], where reduced contrast reaction risk and potential cost savings could be realized. Some authors have made head-to-head comparisons in the shoulder and found equivalent performance for evaluation of glenoid labral and rotator cuff tears, as well as in the detection of acetabular labral tears and cartilage lesions in the hip [25–27].

The intra-articular injection of GBCAs is not approved by the United States Food and Drug Administration (FDA) and is performed as an off-label indication. The small volume administered for dMRA in clinical use is widely considered safe [28], but data from some in vitro studies have suggested that chondrocytes may be adversely affected [29, 30], while others have found no adverse effects [31]. The gadolinium ion does not dissociate from the contrast agent [32], so any ill effects may be attributed to the intact gadolinium chelate [29]. In recent years there has been concern about the deposition of gadolinium into the organs of patients receiving intravenous gadolinium. In one pre-clinical study using rats, detectable levels of gadolinium were present in joint tissues, bone marrow, and/or kidneys following intra-articular injection of both linear and macrocyclic GBCAs, though the clinical significance of this remains unknown [33]. Intra-cranial gadolinium deposition has not been shown after intra-articular administration of GBCAs at clinical doses in either pre-clinical models [33] or on patient brain MRI exams [34, 35].

GBCAs can be diluted over a wide concentration range (0.7–3.4 mmol/L) and still yield acceptable results for dMRA [36], but a concentration range between 1.25 and 2.5 mmol/L is considered ideal for optimum signal-to-noise ratio [37]. To achieve this dilution, one must be aware of the concentration of their particular GBCA, which is summarized in Table 2. The most common commercially available concentration is 0.5 mol/L but may range between 0.25 and 1 mol/L [38]. The addition of iodinated contrast to the mixture allows continuous confirmation of appropriate needle position during the procedure if fluoroscopic guidance is utilized and, if in sufficient amounts (e.g., 25–50% of the total injectate), allows for conversion of dMRA to CT arthrography if necessary [39, 40].

**Table 2:** Concentrations of commercially available gadolinium-based contrast agents

Contrast agent	Structure	Concentration (mol/L)
Dotarem/Clariscan (gadoterate meglumine)	Macrocyclic, ionic	0.5
Eovist/Primovist (gadoxetate disodium)	Linear, ionic	0.25
Gadavist (gadobutrol)	Macrocyclic, nonionic	1.0
Magnevist (gadopentetate dimeglumine)	Linear, ionic	0.5
MultiHance (gadobenate dimeglumine)^1^	Linear, ionic	0.5
Omniscan (gadodiamide)^1^	Linear, nonionic	0.5
ProHance (gadoteridol)	Macrocyclic, nonionic	0.5
Vueway/Elucirem (gadopiclenol)	Macrocyclic, nonionic	0.5

^1^Permitted only for liver imaging in the European Union (EU)

It should be noted that the addition of iodinated contrast results in T1 and T2 signal shortening, and optimum concentrations of gadolinium are lower as a result [36, 37]. This effect is exaggerated at 3 T because the peak signal-to-noise ratios for iodinated contrast dilutions are slightly lower than at 1.5 T [36, 37, 41]. Intra-articular administration of iodinated-based contrast agents is FDA-approved and considered safe, but in vitro studies have suggested chondrotoxicity [30] and transient increases in cartilage stiffness, which may result in an increased risk of tissue or cell damage during weightbearing [42].

Some practitioners include anesthetics in the injectate to provide comfort to the patient during image acquisition [43, 44] or for diagnostic purposes [45], but a growing body of pre-clinical literature points to chondrotoxicity of local anesthetics [46–49]. Limiting intra-articular delivery with use of less chondrotoxic anesthetics (e.g., ropivacaine) may be warranted [46].

### Complications

Although image guided injection of the joints is generally reported to be safe and tolerable by patients [28, 50], a significant number of patients (up to 66%) may experience delayed onset pain in the hours to days following a dMRA [51–53]. Patients under the age of 30 have been reported to experience more pronounced pain, though this can be expected to resolve within a week of the injection [54]. Anaphylactoid reactions may occur after arthrography, including hives (0.4%), with severe anaphylaxis being exceedingly rare (0.003%) [53]. The incidence of joint infection is reported to be 0.003% [55]. Although vasovagal reactions have been reported at a very low rate with dMRA (0.015%), our collective experience is that the frequency is closer to that reported for arthrography in general (1.4%) [53]. Neurovascular complications are exceedingly rare [53], but could occur depending on the chosen needle path. A recent systematic review and meta-analysis has shown that it is safe to perform joint injections in patients on warfarin without routine testing of the international normalized ratio (INR) [56]. Another recent prospective study including 5080 musculoskeletal procedures found no clinically significant bleeding events with warfarin use, continuation of direct oral anticoagulants, or with concomitant antiplatelet or combination antiplatelet therapy [57]. All of these potential risks are important to discuss with patients and document in the consent process.

## Imaging protocols

dMRA has been successfully performed at a variety of field strengths, most commonly 1.5 T. As with cMRI, dedicated extremity coils should be used when available and imaging parameters should be optimized [58, 59]. As with all post-contrast MR imaging at any field strength, short tau inversion recovery is ideally avoided as gadolinium signal may be inadvertently nulled.

The most common sequences that are used with dMRA are T1-weighted fat-suppressed fast or turbo spin echo (FSE/TSE) in all three anatomic planes [3, 60–69]. To better evaluate the bone marrow and soft tissue fat planes, some choose not to fat-suppress one of the planes, while others may add an additional non-fat-suppressed T1-weighted sequence. A fluid-sensitive sequence, such as intermediate-weighted or T2-weighted FSE/TSE with fat-suppression in at least one plane, is also typically included to aid in the evaluation of marrow signal abnormalities and extra-articular structures [54, 60–63, 66–78]. Others recommend 3D gradient (spoiled or steady state) or FSE/TSE sequences with thinner slices, which allow for multi-planar reformats [66, 79–85]. Particularly in the shoulder, some authors have explored the ability to exchange 3D sequences for conventional 2D sequences [86–97]. A recent meta-analysis showed 3D dMRA had similar pooled sensitivity and specificity to 2D dMRA for diagnosing rotator cuff tears and labral lesions, however 3D FSE/TSE sequences demonstrated higher sensitivity than 3D gradient sequences [98].

Metal artifact reduction techniques may be applied with any pulse sequence, including high receiver bandwidth, view angle tilting, and multi-spectral imaging techniques [99–101]. When spectral fat-suppression fails due to non-isocentric positioning or metallic implants, T1-weighted pulse sequences may be obtained without fat-suppression or Dixon techniques may be used.

## “When the dMRA goes wrong”

**Fig. 2:**
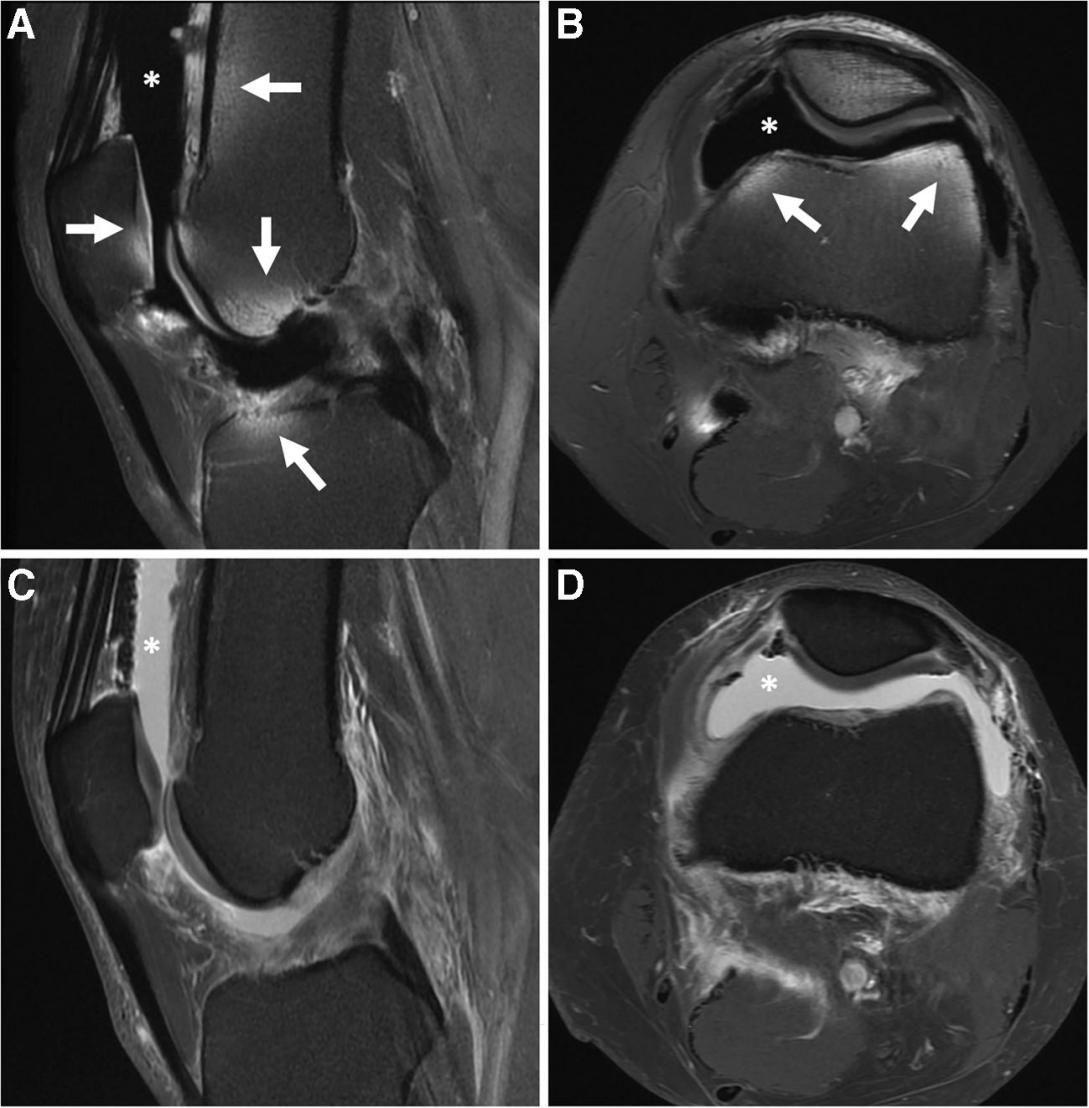
“Black” contrast effect. Thirty-four-year-old man with history of meniscus surgery and persistent knee pain. Sagittal (**A**) and axial (**B**) T1-weighted fat-suppressed 3 T MR images obtained immediately after inadvertent injection of a higher concentration of gadolinium contrast shows hypointense contrast (asterisks). Notice regions of inhomogeneous fat suppression due to the strong paramagnetism of concentrated gadolinium (arrows). Sagittal (**C**) and axial (**D**) T1-weighted fat-suppressed MR images after a 3.5-h delay shows that the majority of the injectate is now hyperintense (asterisks) and there is homogeneous fat suppression

Unexpected issues may arise with improper arthrographic technique. Inadvertent injection of GBCAs outside of the optimal concentration range (e.g., 0.7–3.4 mmol/L [36]) will result in lower signal of the injectate. For concentrations that are too low, standard fluid-sensitive sequences from a cMRI protocol could be used to salvage the exam. For concentrations that are too high, a “black” contrast effect can be seen on the MR images as a result of T2 shortening [102–104]. There is no danger to the patient, and for the larger joints, repeat MR imaging after a few hours can potentially salvage the exam in some instances [102] (Fig. 2). The success of delayed imaging in these scenarios depends on the balance between trans-synovial diffusion of gadolinium and loss of joint distention [23, 105].

**Fig. 3:**
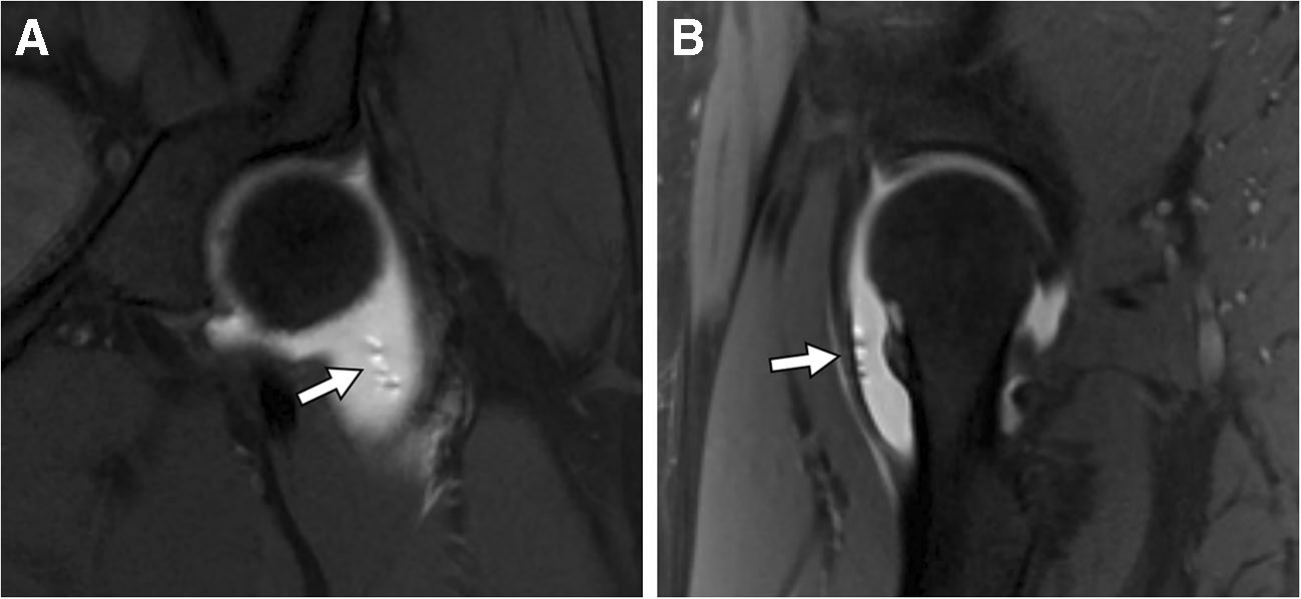
Intra-articular bubbles. Forty-nine-year-old woman with hip pain. Coronal (**A**) and sagittal (**B**) T1-weighted fat-suppressed 3 T MR arthrogram images show bubbles in the nondependent portion of the joint causing a dipole field pattern artifact (arrows)

The unintentional introduction of gas may potentially simulate intra-articular bodies. These bubbles can typically be recognized as they migrate to the nondependent regions of the joint and often demonstrate a characteristic dipole field pattern artifact, with adjacent signal pile-up in one direction and signal loss in the other [61] (Fig. 3). In select cases where pathology is simulated, the patient can be reimaged in a prone position to mobilize the foci of gas to another nondependent location.

There are instances where the injectate may be in an unexpected location. For this reason, it is prudent for the radiologist to review the initial images (i.e., after the localizers or first series is completed), or if possible, request a notification from the technologist for any unusual patterns of contrast distribution. For cases where the injectate is entirely outside of the joint, such as into a bursa (e.g., subacromial-subdeltoid bursa in the shoulder or iliopsoas bursa in the hip), extra-synovial fat pad, or in a recess that happens to be separated from the joint (e.g., cases of complete suprapatellar plica), completing the MRI exam using a routine non-contrast protocol would be advised. It is optimal to empathetically disclose this ‘maloccurrence’ to the patient at the time of imaging. If the patient agrees and if time allows, a repeat injection could be performed prior to completing the MR examination. Alternatively, the patient can be requested to return for a repeat procedure.

Rarely the dMRA exam may not be completed due to MRI scanner failure, or a scan may be severely compromised or even aborted due to patient motion and/or intolerance. In these instances, conversion to CT arthrography may be considered if iodinated contrast was included in the injectate. In one study where CT arthrography of the knee was performed an average of 100 min following a dMRA injection (a scenario that mimics the delay that may be experienced with an aborted exam), accuracy for meniscal tears remained high [62].

## Pathology-specific, joint independent indications

### Chondral and osteochondral abnormalities

**Fig. 4:**
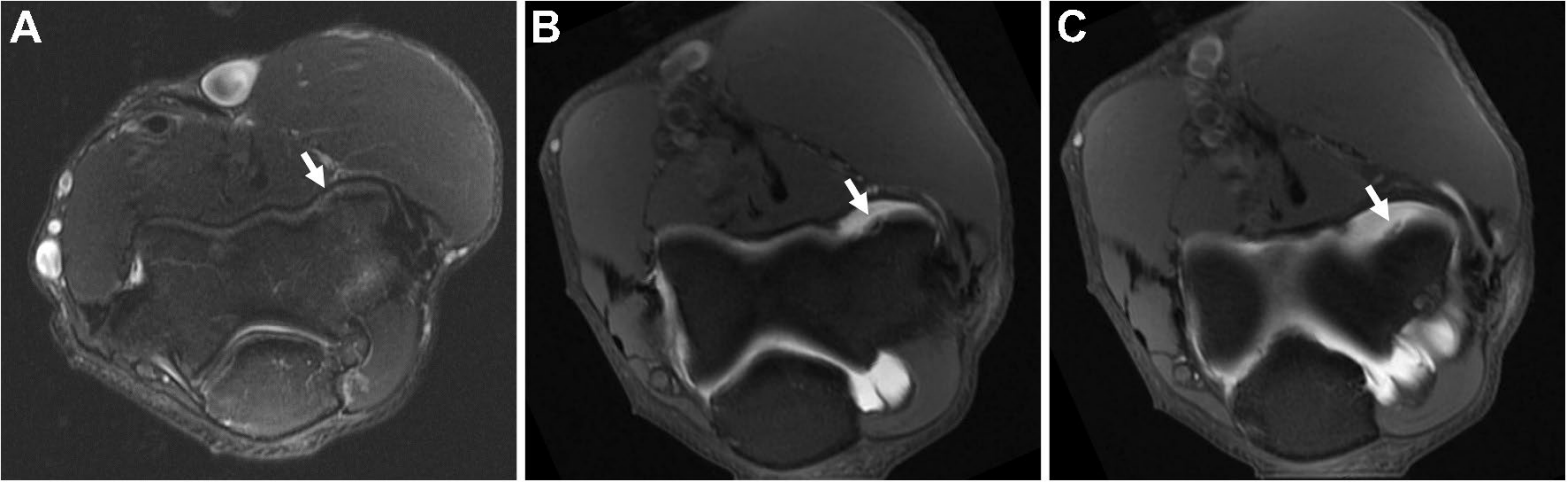
Osteochondral lesion. Thirty-eight-year-old man with elbow pain. **A** Axial T2-weighted fat-suppressed conventional 1.5 T MR image shows a small osteochondral lesion at the anterior capitellum, without signs of instability (arrow). **B**, **C** Axial T1-weighted fat-suppressed 1.5 T MR arthrogram images show contrast undermining the lesion, consistent with an unstable in situ osteochondral fragment

**Fig. 5:**
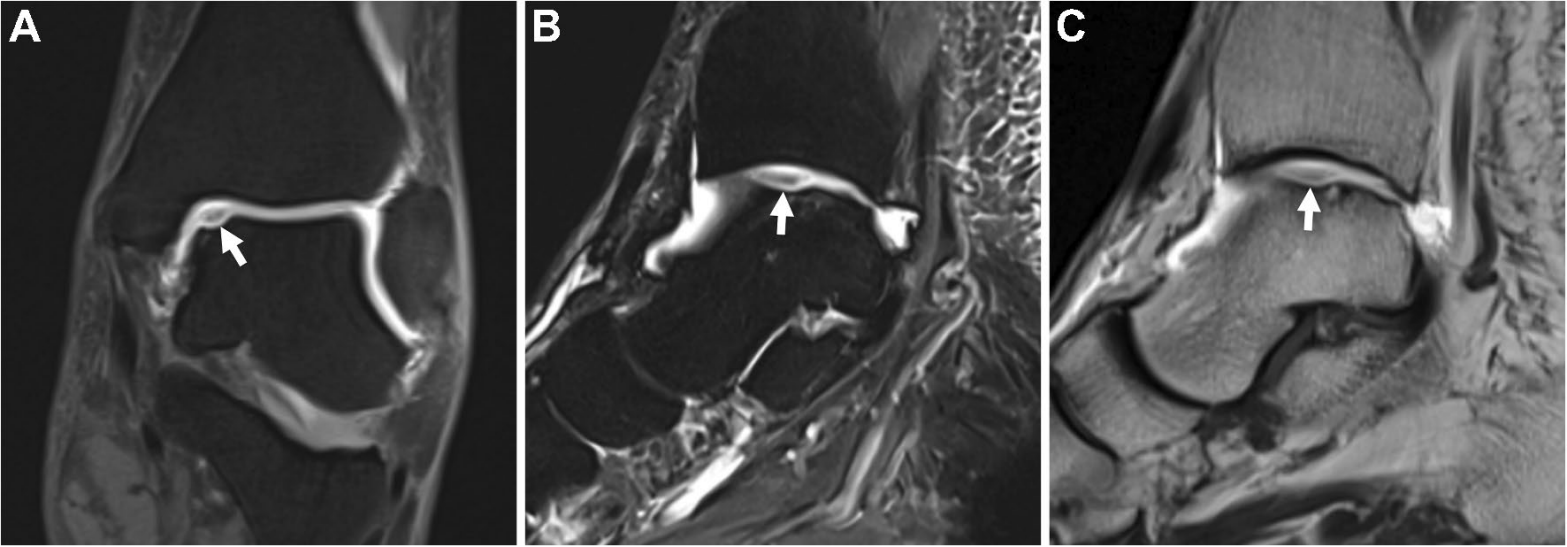
Osteochondral lesion. Sixty-two-year-old woman with ankle pain. Coronal T1-weighted fat-suppressed (**A**), sagittal STIR (**B**), and sagittal T1-weighted 3 T MR arthrogram images demonstrate an osteochondral lesion at the medial aspect of the talar dome with intra-articular contrast that insinuates into the interface with the talus (arrows), consistent with instability

Direct MR arthrography may be useful to diagnose and stage chondral and osteochondral injuries [28]. The differentiation between a stable and unstable nondisplaced in-situ fragment is important for surgical decision-making. Overlapping imaging appearances exist between stable and unstable in situ osteochondral fragments, but fragment instability can be confidently diagnosed when fluid (either native or introduced via dMRA) is seen extending along the entirety of the interface between the fragment and underlying tissue [28] (Figs. 4 and 5).

There is a paucity of head-to-head comparison studies between cMRI and dMRA in the shoulder, elbow, wrist, and ankle regions. In the shoulder, dMRA has shown moderate diagnostic performance, due to reduced sensitivity, for detecting glenohumeral cartilage lesions with fair to moderate interobserver agreement [106–108]. In the ankle, reported accuracies for diagnosis and grading of osteochondral lesions of the talus appear comparable between cMRI and dMRA [109–112].

In the elbow, published algorithms for evaluating osteochondral lesions in adolescent athletes include radiographs and cMRI, particularly for establishing lesion stability and to guide surgical management [113, 114]. Despite the lack of studies directly comparing cMRI and dMRA, several studies have demonstrated a high sensitivity of cMRI for the detection of unstable osteochondral lesions, relying primarily on the visualization of a high, fluid-signal intensity rim undermining the interface between the lesion and underlying bone and a full-thickness articular cartilage defect [115, 116]. One study comparing cMRI to a gold standard of arthroscopy in 52 osteochondral lesions found 100% sensitivity and 80% specificity for unstable lesions and close correlation in 94% of cases with the International Cartilage Repair Society classification for lesion instability [117]. In contrast, a recent meta-analysis concluded that cMRI criteria for instability are adequate for assessing adult osteochondral lesions of the elbow, knee, and ankle, but performed less well for predicting stability in pediatric osteochondral lesions [118]. This meta-analysis included 3 studies using cMRI for the assessment of pediatric elbow osteochondral lesions. The collective sensitivity, specificity, and accuracy from these studies can be calculated as 94%, 66%, and 84% respectively.

At the hip, dMRA performs more favorably compared with cMRI. cMRI of the hip is known to have limited sensitivity but high specificity in the detection of chondral abnormalities [119]. A meta-analysis of the accuracy of MR imaging in the detection of chondral lesions in the setting of femoroacetabular impingement (FAI) showed sensitivity, specificity, and accuracy of 76%, 72%, and 75% respectively for cMRI, compared to 75%, 79%, and 83% for dMRA [120]. This analysis included studies which utilized magnetic field strengths ranging from 1 to 3 T [120]. However, a subsequent study suggested that 3T cMRI may be superior to 1.5 T dMRA for the detection of cartilage lesions, but the differences were small and should be interpreted with caution [121]. In that same study, when specifically assessing cartilage delamination, 3 T MRI was equivalent to 1.5 T MR arthrography [121]. Another study compared 3T cMRI to dMRA in the detection of acetabular chondral defects in the same patient population and suggested greater sensitivity with dMRA (sensitivity/specificity of the two readers: 65%/100% and 59%/100% for cMRI compared to 81%/91% and 71%/82% for dMRA), although the differences were not significant [122]. A study which specifically addressed chondral delamination in the setting of FAI found a sensitivity of 6%, specificity of 98%, NPV 27% and PPV 91% with 1.5 T dMRA [123]. Attempts have been made to improve the conspicuity of hip cartilage lesions, including delamination, using leg traction at the time of dMRA [124]. Although preliminary experience suggested that traction does indeed aid in detecting surface cartilage lesions and cartilage delamination, the literature has shown mixed results [123, 125, 126]. In addition, the time involved and fear of potential patient discomfort have dissuaded most sites from employing hip traction at the time of MR imaging.

At the knee, dMRA has performed favorably compared with cMRI. In one meta-analysis, dMRA was found to be superior to conventional MRI for detection of patellofemoral chondral lesions [127]. For studies that have compared dMRA to cMRI for cartilage abnormalities in the knee, sensitivities have ranged from 69 to 93% and specificities have ranged from 98 to 100% for dMRA, while sensitivities have ranged from 25 to 81% and specificities have ranged from 50 to 99% for cMRI [65, 84, 128–131]. It is noteworthy that both dMRA and cMRI are less accurate for grade I compared with grade IV cartilage abnormalities [65, 128, 130], but delayed image acquisition by a few hours may improve the performance of dMRA for grade I lesions [22, 84, 132]. For osteochondral lesions in 25 knee MRI exams, one study found that correct staging could be performed in 100% of cases on dMRA and 57% of cases on cMRI [133].

*Recommendation: dMRA or cMRI recommended.* The use of dMRA may be valuable when symptoms are discrepant with cMRI results (*consensus recommendation, unanimous*).

### Post-operative evaluation of chondral and osteochondral abnormalities

A variety of chondral and osteochondral restoration techniques exist, including marrow stimulation, osteochondral transplantation (autologous or allogeneic), autologous chondrocyte implantation, and allogeneic particulate cartilage fragment implantation [134]. Direct MRA may be useful to delineate defects at the cartilage interface following all types of repair or graft/host bone junction following osteochondral repairs [135–137], but the protocol should include fluid sensitive and non-fat-suppressed sequences to assess the subchondral marrow and trabeculae. Authors have used dMRA to evaluate patients after matrix-induced autologous chondrocyte implantation in mid- and long-term follow-up studies [63, 74], but one study found that the dMRA findings correlated poorly with clinical outcomes [74].

Other authors have reported effective evaluation of chondral repair tissue without the use of dMRA [138, 139] and, in fact, cMRI is most widely used to assess the features deemed the most important after chondral repair or osteochondral transplantation [140–142].

*Recommendation: dMRA or cMRI recommended (consensus recommendation, supermajority (10/12)).*

### Intra-articular bodies

Intraarticular bodies may present without any history of prior injury and may cause limited range of motion, pain, catching or locking [143, 144]. Imaging can confirm the diagnosis and provide useful surgical planning information [145, 146]. When ossified, intraarticular bodies can be visualized by radiography, however radiography is frequently inadequate [144, 147, 148]. cMRI often detects an intraarticular body, especially with a joint effusion; however, dMRA may prove useful in detection of small bodies [28, 60, 143, 145, 149].

There is a paucity of head-to-head comparison studies between cMRI and dMRA in the shoulder, elbow, wrist, and ankle regions. In the hip, one study showed high diagnostic accuracy for the detection of intra-articular osteochondral bodies using dMRA, both without and with traction [150]. Using osseous and cartilaginous bodies placed inside 16 cadaveric knees, one study showed 92% accuracy for detection using dMRA compared with 57–70% using cMRI [151].

*Recommendation: cMRI recommended (consensus recommendation, supermajority (11/12)).*

## Joint-specific indications

### Shoulder

#### Technical considerations

The recommended target for fluoroscopic glenohumeral joint injection varies, with many advocating the medial upper third of the humeral head and others advocating the middle or lower third [152–155]. One study reported an 85% first attempt success rate utilizing the rotator interval approach for resident trainees and 100% success rate with reduced fluoroscopy time for musculoskeletal radiologists [153]. Additional techniques using a posterior approach have been proposed to avoid traversing the anterior stabilizing structures and confounding imaging findings related to inadvertent extra-articular contrast leakage [156, 157]. Comparison of US-guided rotator interval and posterior approach injections found that both techniques are successful and well tolerated by patients, but the posterior technique resulted in decreased extra-articular leakage rate [158, 159].

The volume of injectate used for direct shoulder MR arthrography varies in the literature, ranging from 8 to 15 ml, with some authors titrating to perceived resistance to the injection while monitoring distention with imaging [152, 155]. A patient with chronic capsular laxity may have a higher injection capacity, whereas a patient with adhesive capsulitis may tolerate a smaller volume injectate [160–163]. In work with cadavers, a volume of 15 mL of intraarticular fluid has been described as optimal for dMRA, but little data exists comparing adequate joint distention with rates of contrast leakage [164]. The authors recommend a minimum of 8 ml of injectate. After shoulder arthrography, neither internal derangements nor history of prior surgery had an apparent effect on the post-injection pain course, and post-injection exercise prior to MRI does not improve image quality or the depiction of rotator cuff or labral tears [165, 166].

#### Provocative maneuvers

**Fig. 6:**
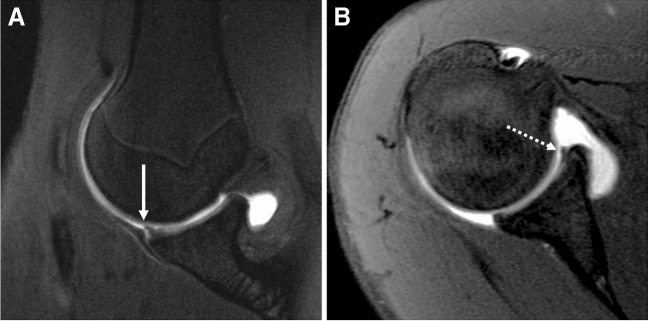
Abduction external rotation (ABER) positioning. Sixteen-year-old with history of recent shoulder dislocation. **A** T1-weighted fat-suppressed MR arthrogram image in the ABER position shows a tear of the anterior labrum which remains partially attached (arrow), consistent with a Perthes lesion. **B** On conventional axial T1-weighted fat-suppressed image, the tear is less evident (dashed arrow)

In specific clinical instances, an additional fat suppressed T1-weighted abduction external rotation (ABER) sequence may be performed to obtain an oblique axial plane with regard to the glenohumeral joint [167]. The ABER position changes the capsular dynamics by creating traction on the anterior band of the inferior glenohumeral ligament and axillary pouch structures, which may increase the conspicuity of anteroinferior and posterosuperior labral lesions [167–169] (Fig. 6). The total additional time for patient re-positioning and scanning ranges from approximately 5–15 min for the ABER sequence [170–172]. Patients may be apprehensive to assume the ABER position due to shoulder pain or concern for dislocation resulting in increased motion artifact, but one study reports a 95% success rate through patient education combined with lidocaine in the intra-articular injectate [167, 172, 173]. However, some centers elect to forgo the ABER sequence due to practical concerns about throughput as well as consistency and reproducibility across sites*.* A recent survey of European Society of Musculoskeletal Radiology (ESSR) members reported the ABER sequence is used in about half of cases, with no differences between general (60%) and orthopedic (65%) hospitals [174].

The ABER sequence has comparable sensitivity and specificity to conventional dMRA for anteroinferior labral lesions [170, 171, 175, 176]. Several studies have shown no significant differences in interobserver agreement between the neutral and ABER position [170, 171]. A recent meta-analysis showed increased diagnostic accuracy of routine axial plus ABER dMRA (pooled sensitivity 95.7%, pooled specificity 94.5%) compared with dMRA without ABER (pooled sensitivity 81.5%, pooled specificity 88.8%). However, results are interpreted with caution since the 95% confidence intervals overlapped, there was a high degree of heterogeneity among the studies, and there was publication bias for axial plus ABER dMRA studies [172]. Furthermore, of the 9 articles included in the meta-analysis, 3 articles were at least 20 years old, and 2 additional articles included cases that were over 20 years old. All considered, the superiority of ABER over routine axial positioning, when scanning using modern imaging technology would benefit from further study, and ideally would consider relative time on the MRI scanner/cost considerations. When evaluating only studies performed at 3 T, another meta-analysis showed improved sensitivity for the diagnosis of anterior and posterior labral lesions with dMRA over cMRI, including improved sensitivity but reduced specificity with ABER positioning for anterior labral tears [177] . In particular, the ABER sequence may add value in the detection of the Perthes variant of anteroinferior labral lesions and after Bankart repair [171, 178, 179]. However, ABER sequences have been reported to have decreased accuracy in the characterization of anterior labroligamentous periosteal sleeve avulsion (ALPSA) lesions [171, 180]. For SLAP lesions, there is no definitive data to support increased accuracy with inclusion of the ABER sequence [181, 182], though it has been suggested to be useful in characterizing posterosuperior labral peel back lesions in throwing athletes [183]. Assessment of combined redundancy signs during ABER positioning has been advocated to differentiate patients with atraumatic multi-directional instability (MDI) from clinically stable shoulders with 81-90% sensitivity and 94% specificity [184].

Additional specialized provocative maneuvers have been described, but thus far, have not been adopted at most centers for routine clinical use. Weighted traction and FADIR (flexion, adduction, internal rotation) positioning may increase the conspicuity of SLAP tears and posteroinferior labral lesions respectively [185, 186]. ADIR (adduction internal rotation) positioning has been reported to improve characterization of ALPSA lesions, but data is limited [180].

*Recommendation*: Provocative maneuvers are *not recommended* in routine practice. Selective application of imaging in the ABER position may be advantageous in some patients with anterior instability. This decision is ideally made when an anteroinferior labral abnormality is not identified on review of the routine axial images in a patient with known anterior instability **(***consensus recommendation, majority (9/12)***).**

#### Clinical indications

##### Instability

**Fig. 7:**
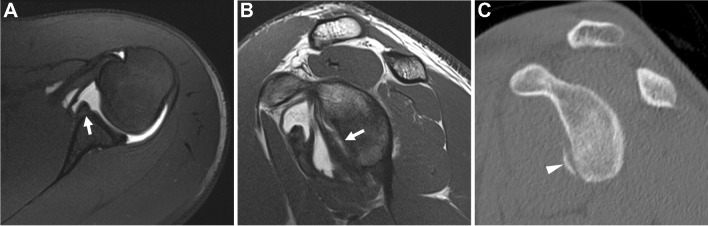
Anterior instability. Twenty-year-old man with recent anterior glenohumeral dislocation while playing rugby. Axial T1-weighted fat-suppressed (**A**) and sagittal T1-weighted (**B**) 1.5 T MR arthrogram images demonstrate a mixed fibrocartilaginous and osseous Bankart variant lesion with deficiency of the anterior glenoid (arrows). **C** Sagittal reformat image from CT scan 4 months later demonstrates the displaced osseous Bankart component (arrowhead)

Anterior instability is one of the most common indications for dMRA [174, 187]. One study demonstrated 88% sensitivity, 91% specificity, and 89% accuracy with regard to dMRA and detection of anteroinferior labral tears [188] (Fig. 7). More recently, pre-operative dMRA has been reported as a predictor of recurrent instability based on calculation of “off-track bone loss” [189].

**Fig. 8:**
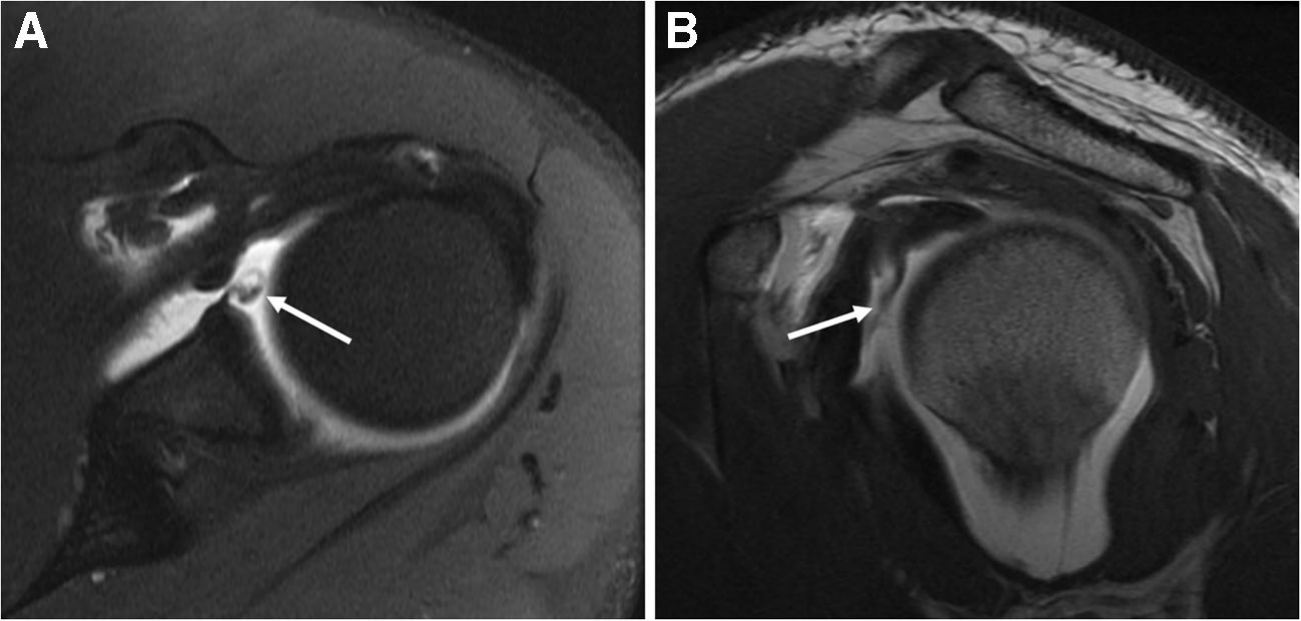
Microinstability (type VI SLAP). Eighteen-year-old man with clinically suspected labral tear. Axial T1-weighted fat-suppressed (**A**) and sagittal T1-weighted (**B**) MR arthrogram images show a tear of the anterosuperior labrum with a fragment displaced into the anterosuperior joint recess (arrows)

Using arthroscopy as the reference standard, dMRA showed a sensitivity of 82% and specificity of 98% in the identification of superior labrum anterior and posterior (SLAP) tears [190] (Fig. 8). Multiple meta-analyses have shown that dMRA is more accurate than cMRI for the diagnosis of SLAP tears [181, 182, 191, 192], with higher diagnostic accuracy at 3 T over 1.5 T with or without intra-articular contrast material [181]. One meta-analysis evaluating 3 T studies showed that dMRA was similar in sensitivity to cMRI (0.84 vs 0.83, p=.575), but less specific (0.99 vs 0.92 *p* < 0.0001) for SLAP lesions [177].

**Fig. 9:**
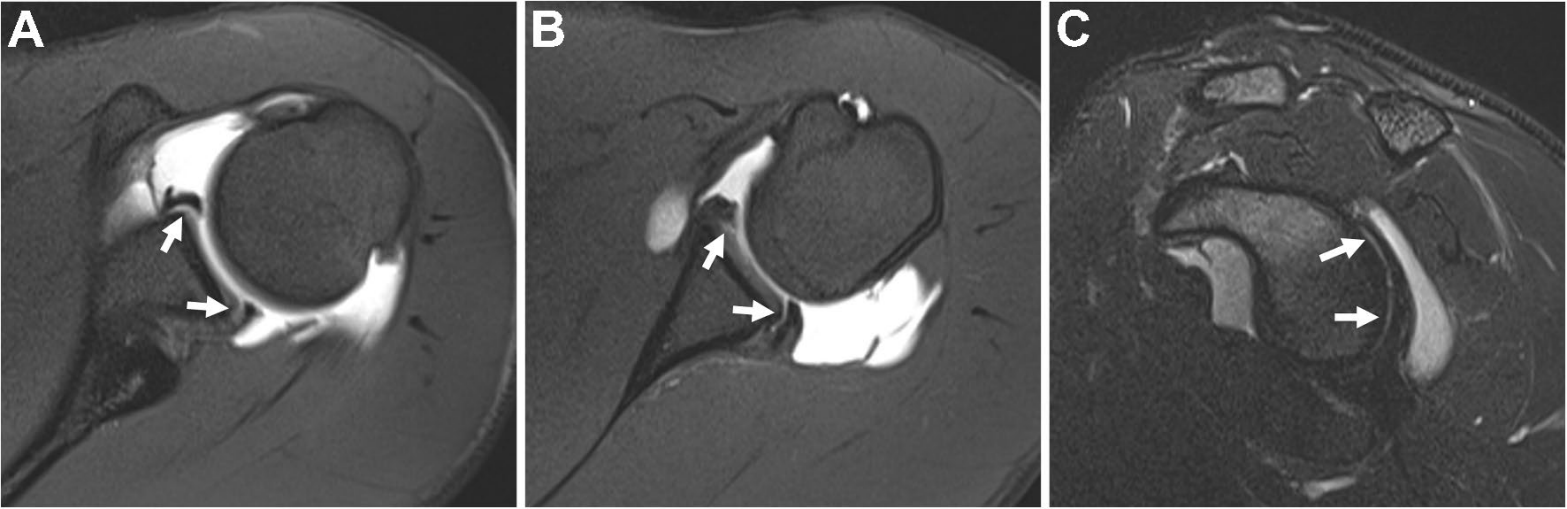
Multidirectional instability. Eighteen-year-old man with shoulder pain. Axial T1-weighted fat-suppressed (**A** and **B**) and sagittal T2-weighted fat-suppressed (**C**) 1.5 T MR arthrogram images demonstrate extensive labral tearing involving both anterior and posterior aspects (arrows)

Direct MR arthrography has been proposed as a method to aid the complex clinical diagnosis of multidirectional instability (MDI) (Fig. 9). Prior studies have shown an increased capsular volume on dMRA in MDI patients [160, 193]. In particular, greater inferior and posteroinferior axillary recess depths have been accurate and reproducible in differentiating MDI from control groups [161, 193–195]. The rotator interval width may be greater in patients with other forms of instability but remains debated in regard to diagnostic utility for MDI [160, 161, 184, 193, 195–197].

*Recommendation: dMRA or cMRI recommended.* Direct MRA has a compelling role in the assessment of younger individuals with suspected instability, when subtle labroligamentous abnormalities may have profound influences on shoulder function, management, and prognosis **(***consensus recommendation, unanimous***)**.

##### Humeral avulsion of the glenohumeral ligament (HAGL)

**Fig. 10:**
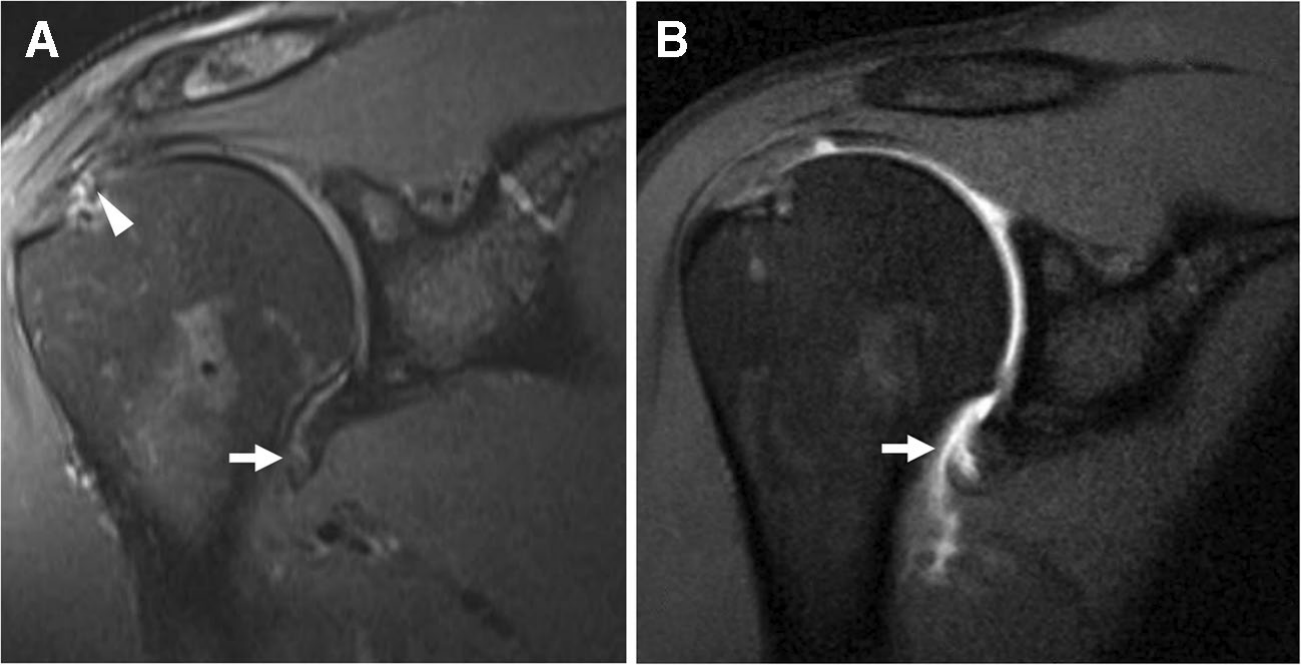
Chronic humeral avulsion of the glenohumeral ligament (HAGL). Twenty-seven-year-old professional pitcher with shoulder pain. **A** Coronal intermediate-weighted fat-suppressed conventional 1.5 T MR image shows irregularity of the inferior glenohumeral ligament (IGHL) complex (arrow) and partial-thickness tearing of the supraspinatus tendon (arrowhead). **B** Coronal T1-weighted fat-suppressed 1.5 T MR arthrogram image shows extra-articular contrast leakage and a thickened, retracted IGHL margin, consistent with a HAGL

Direct MRA may be helpful in diagnosing inferior glenohumeral ligament (IGHL) injuries. Extra-articular contrast material leakage and the J-sign are described imaging signs [198, 199] (Fig. 10). However, iatrogenic extra-articular contrast leakage at dMRA is not uncommon and should be interpreted with caution [200, 201]. A recent study examined several dMRA features that can be used to distinguish iatrogenic extra-articular contrast leakage and true IGHL tears [202]. There are no studies directly comparing the diagnostic accuracy of cMRI and dMRA for HAGL injury.

*Recommendation: dMRA or cMRI recommended (consensus recommendation, unanimous).*

##### Thrower’s shoulder

**Fig. 11:**
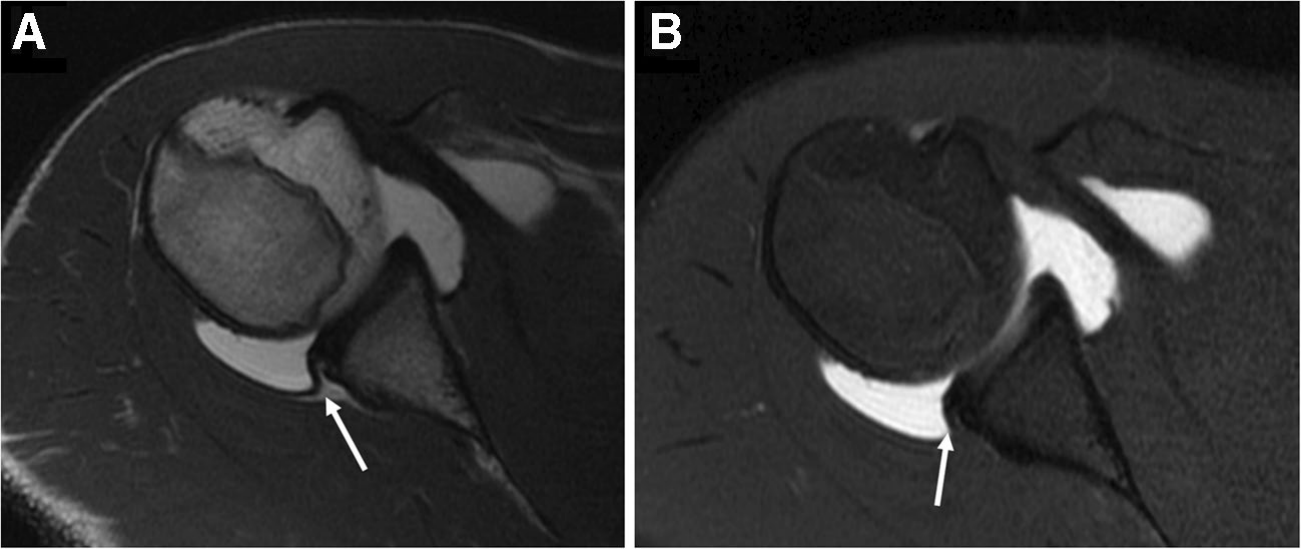
Thrower’s shoulder. Fifteen-year-old baseball player with persistent right shoulder pain. Axial intermediate-weighted (**A**) and T1-weighted fat-suppressed (**B**) MR arthrogram images show a thickened posterior band of the inferior glenohumeral ligament at the labral insertion (arrows) with associated glenoid remodeling and retroversion. Constellation of imaging findings was consistent with the clinical diagnosis of glenohumeral internal rotation deficit (GIRD), and the patient was treated conservatively with physical therapy

High-performance athletes may represent a subgroup of patients for whom initial dMRA is indicated as it potentially yields more diagnostic information over cMRI, considering the greater sensitivity for partial-thickness articular-sided rotator cuff tears and posterosuperior labral pathology [203–205] (Fig. 11). In throwing athletes, dMRA may also assist in characterizing biceps pulley lesions resulting in anterosuperior impingement as well as internal impingement and glenohumeral internal rotation deficit (GIRD) [206, 207]. It should be noted that these cited studies were mostly published prior to 2007 with imaging performed at 1.5 T.

*Recommendation: dMRA or cMRI recommended (consensus recommendation, unanimous).*

##### Recurrent instability and the post-operative labrum

**Fig. 12:**
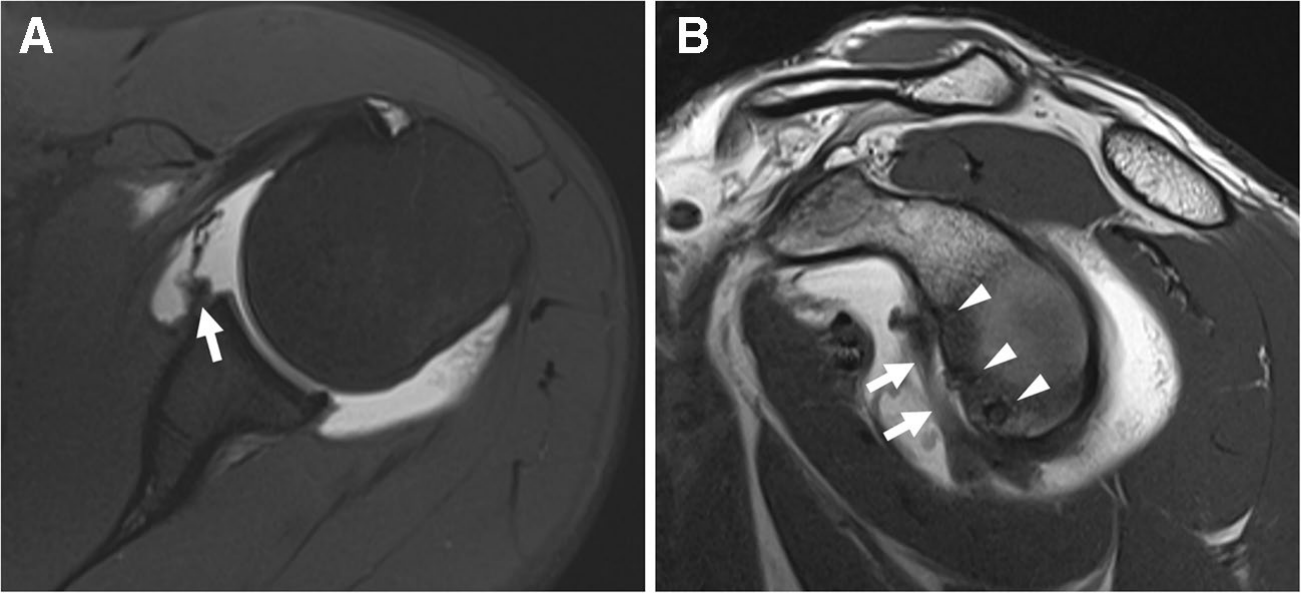
Recurrent anterior labral tear. Twenty-six-year-old man with anterior glenohumeral dislocation 1 year following arthroscopic Bankart repair. Axial T1-weighted fat-suppressed (**A**) and sagittal T1-weighted (**B**) 1.5 T MR arthrogram images demonstrate medialized and scarred anteroinferior labroligamentous tissue (arrows), consistent with a chronic anterior labroligamentous periosteal sleeve avulsion (ALPSA). Note the anterior glenoid anchor tracks (arrowheads)

Shoulder imaging following surgery for instability can present as a challenge secondary to scar tissue, and these patients are likely to benefit from dMRA (Fig. 12). Using arthroscopy as the gold standard, one author found that sensitivity for labral and supraspinatus tears after repair ranged from 71 to 84% with cMRI and increased to 100% with dMRA at 3 T [40]. In another study using dMRA at 1.5 T, the accuracy for labral tears (anterior, posterior and SLAP tears) was 92% and comparable to dMRA of the native shoulder [208]. Another study that compared dMRA (neutral and ABER) at 1T to second look arthroscopy correctly confirmed the structural integrity of the repaired glenoid labrum with an accuracy of 95% [179].

*Recommendation: dMRA recommended (consensus recommendation, unanimous).*

##### Rotator cuff tear and re-tear

Several meta-analyses and systematic reviews have concluded dMRA as the most sensitive imaging method for detection of rotator cuff tears when including all field strengths [209–212], but the diagnostic advantage is less pronounced for full-thickness tears [209–211]. dMRA at 3 T shows similar sensitivity to dMRA at 1.5 T as well as similar specificity to 3 T cMRI for the diagnosis of full-thickness tears, but improved sensitivity for partial-thickness tears [209, 213]. As expected, dMRA performs slightly better than cMRI for articular sided partial-thickness tears (sensitivity 74% and specificity 90% for dMRA versus sensitivity 67% and specificity 82% for cMRI) but shows similar sensitivity (0.75 dMRA versus 0.73 for cMRI) for detection of bursal-sided tears [209, 214]. However, it should be noted that both of these studies specifically addressing bursal surface tear detection included indirect MRA in the subgroup analysis for partial-thickness tears [209, 214].

Regarding subscapularis tears, there is limited data on the best diagnostic method. Both dMRA and cMRI have shown relatively lower accuracy in the diagnosis of subscapularis tears, due to decreased sensitivity, particularly for partial thickness tears [209, 213, 215–217]. Studies have suggested that dMRA may be marginally better for subscapularis tears at 3 T [213, 216]. 2D and 3D dMRA at 3 T appear to be equivalent for all types of rotator cuff tears [98, 213]. Regarding recurrent rotator cuff tears, there are relatively few studies assessing the diagnostic accuracy of dMRA when surgical correlation is used as the reference standard [218–220]. One study reported high sensitivity (88%) and specificity (90%) for full-thickness tears, but overall moderate sensitivity (72%) and specificity (77%) for partial-thickness tears and only fair interobserver agreement for subscapularis tears (k = 0.20) [218]. A recent meta-analysis had insufficient data to separate dMRA from cMRI in subgroup analyses to determine the most accurate imaging method [220].

The ABER sequence has been reported to have similar sensitivity and specificity for full-thickness superior rotator cuff tears with good interobserver reproducibility [170, 221] but may improve the sensitivity for detection and characterization of partial-thickness articular surface tears [169, 222–224].

*Recommendation: cMRI recommended* as the differences in sensitivity and specificity between dMRA and cMRI for the diagnosis of rotator cuff tear and retear are small **(***consensus recommendation, supermajority (11/12)***)**.

##### Adhesive capsulitis (AC)

Several MR imaging signs of AC have been reported [225]. The majority of studies examining the utility of dMRA for the diagnosis of AC compare clinically symptomatic patients to asymptomatic control subjects or those with other shoulder pathology [163, 226–229]. Few studies report surgical or arthroscopic confirmation in at least a portion of patients [71, 230, 231]. Studies comparing patients with a clinical diagnosis of AC to controls have found thickening of the rotator interval joint capsule and coracohumeral ligament as suggestive for AC [71, 227]. Reliance on visualization of axillary pouch abnormalities on dMRA alone is unclear as there is conflicting data with respect to the utility of axillary recess capsular thickening and diminished filling capacity [71, 226–229, 231–233]. Complete obliteration of the subcoracoid fat triangle has poor sensitivity but high specificity for adhesive capsulitis; however, coracohumeral ligament and IGHL thickening have a higher correlation with range of motion impairment [71, 227, 232]. The utility of measuring the rotator interval dimensions on dMRA is also limited with conflicting evidence [163, 230, 232]. On 3D volumetric assessment, decreased joint capacity has also been reported with greater frequency in AC patients versus control subjects [163]. One systematic review and meta-analysis recommended cMRI over dMRA in AC as the sensitivity and specificity of IGHL thickening on cMRI and dMRA were not significantly different from one another [234].

*Recommendation: cMRI recommended (consensus recommendation, unanimous).*

##### Long head biceps tendon (LHBT) and pulley lesions

Similar to cMRI, dMRA is insensitive for LHBT tendinopathy, particularly when compared with histopathology [235–240]. When images are evaluated in two planes, dMRA is reasonably accurate for diagnosing biceps rupture [240]. Comparison of 1.5 T cMRI and dMRA in 199 patients who underwent arthroscopy, the authors found no significant difference between the two methods for the detection of intra-articular LHBT tendinosis and tears [238]. At 3 T, dMRA was insensitive for biceps partial-thickness tears and performed similar to CT arthrography [239]. Diagnosing LHBT instability on static images can be challenging in the absence of frank tendon dislocation. Individual imaging findings have a limited role in diagnosing LHBT instability, but the accuracy of dMRA can be improved by combining imaging findings and assessing the integrity of the biceps pulley structures [235, 241–245]. In cadavers, dMRA was superior to cMRI for evaluation of the rotator interval structures [246]. For considerations specific to throwing athletes, please refer to the “Thrower’s shoulder” section.

*Recommendation: cMRI recommended (consensus recommendation, supermajority (10/12)).*

### Elbow

#### Technical considerations

Initially, the lateral radiocapitellar needle approach was described due to ease of radial head palpation, but also to avoid contrast within the medial capsule, which was often injured [247]. For this method, the anterior half of the radiocapitellar joint is targeted with the patient prone and the arm extended above the head in 90° of elbow flexion [12, 248]. For patients who cannot lie prone, they are seated with the arm abducted, flexed 90°, and resting on the fluoroscopy table [12]. Another common method is a posterior transtriceps approach [249]. Patients are positioned similarly, though a posterior needle placement is chosen centered between the epicondyles with the needle aimed to the olecranon fossa, which provides a backstop for needle depth [249–252]. In a retrospective study comparing these two methods, the posterior approach resulted in less extra-articular contrast leakage and decreased cases with diagnostic dilemma [252]. A less common approach is posteromedial [248, 253]. Although injection volume varies from 6 to 10 mL [253], one study reported finding that 6 of 7 patients with volume greater than 8 mL had moderate extra-articular contrast leakage, while 6 of 12 patients with less than 8 mL of injectate showed minimal to no contrast leakage [250]. We recommend 3–6 mL of contrast injection to avoid extraarticular contrast. Traction has not proven beneficial in the few studies that have evaluated it [254, 255].

#### Clinical indications

##### Posterolateral rotatory instability (PLRI)

Conventional MR imaging provides direct visualization and high accuracy for evaluation of the lateral ulnar collateral ligament (LUCL) [256, 257]. In a cadaveric study, cMRI (high-resolution intermediate-weighted images) showed greater performance and higher reader agreement compared with dMRA [258]. Despite this, some orthopedic surgeons prefer dMRA evaluation [259]. In the future, 3D isotropic MR imaging may provide improved LUCL assessment [260].

*Recommendation: cMRI recommended (consensus recommendation, supermajority (11/12)).*

##### Valgus instability/thrower’s elbow

**Fig. 13:**
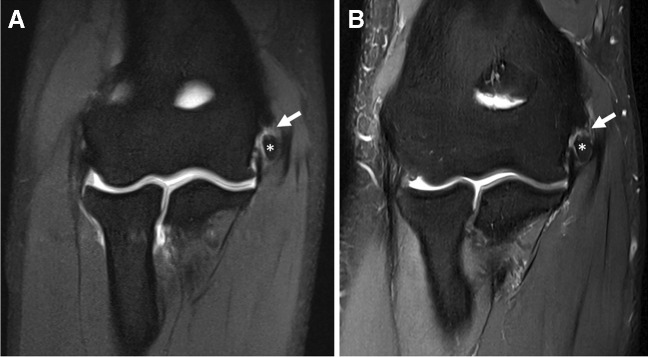
Thrower’s elbow. Twenty-two-year-old man with medial elbow pain and suspected ulnar collateral ligament tear. **A** Coronal T1-weighted fat-suppressed MR arthrogram image at the time of injury shows an ulnar collateral ligament tear at the humeral attachment (arrow) with adjacent ossicle (asterisk). **B** Coronal intermediate-weighted fat-suppressed conventional 3T MR image three months later shows the tear (arrow) and ossicle (asterisk) to similar advantage

Direct MR arthrography has been reported to provide a higher sensitivity and specificity compared to cMRI for evaluation of ulnar collateral ligament (UCL) tears, especially in elite athletes, with the additional advantage for identifying undersurface partial-thickness tears [24, 261–265]. However, dMRA has a lower sensitivity for proximal UCL tears (64%) [263], which comprised 48% of total tears and 42% of high grade tears in one large series [266] (Fig. 13). Although no imaging comparison between dMRA and cMRI has been reported in the postoperative setting, one study suggests that dMRA helps delineate thickening and intermediate graft signal with recurrent tears [265]. However, prediction of valgus laxity is inaccurate by both cMRI and dMRA, which requires dynamic/stress examination. Where clinically feasible, combining dMRA with stress US evaluation may prove useful [263]. In throwing athletes, the addition of a flexed elbow valgus external rotation (FEVER) positioning on cMRI resulted in increased diagnostic confidence and additional UCLs that were identified as abnormal [267].

*Recommendation: dMRA or cMRI recommended (consensus recommendation, majority (9/12)).*

##### Plica

**Fig. 14:**
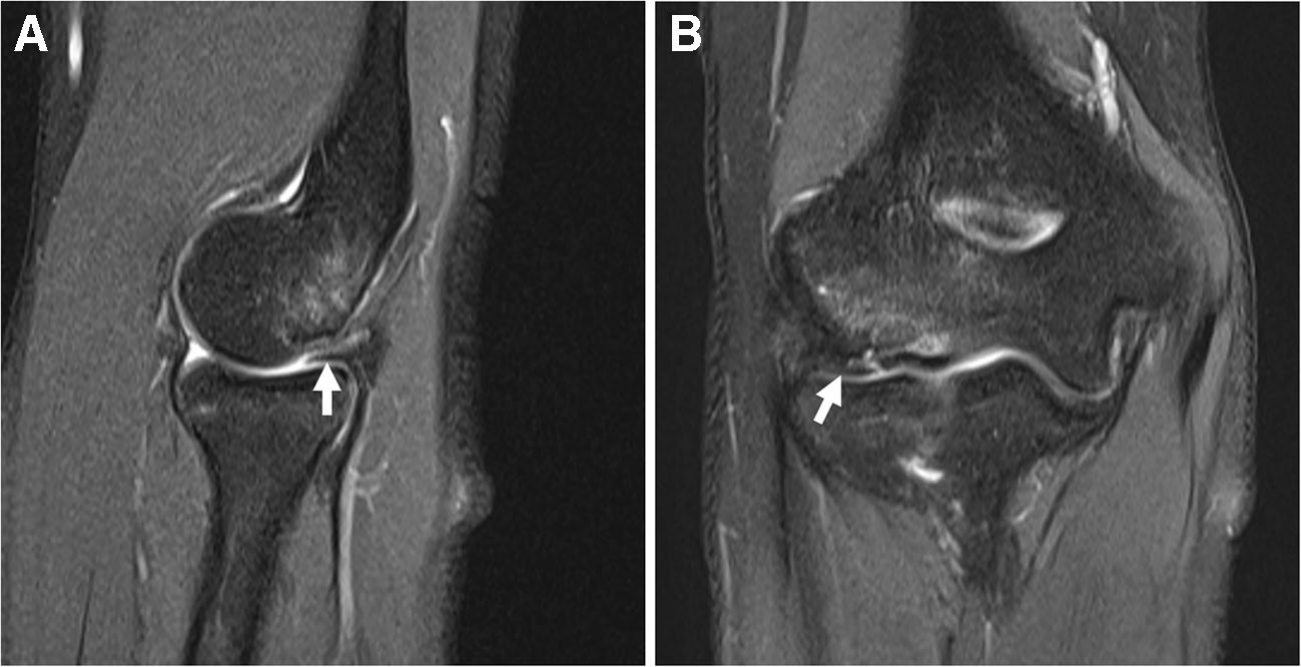
Plica syndrome. Seventeen-year-old elite volleyball player with posterolateral elbow pain. Sagittal (**A**) and coronal (**B**) intermediate-weighted fat-suppressed conventional 1.5 T MR images demonstrate a thickened radiocapitellar plica (arrows) with adjacent bone marrow edema, consistent with the clinical diagnosis of impingement. The plica was arthroscopically resected with resolution of symptoms

Direct MR arthrography may be useful for evaluating elbow plica. Plicae are synovial fold remnants from embryonic development that serve no known purpose but can inflame with repetitive trauma and result in pain, catching or locking [268, 269]. Plica occur in several locations with the posterolateral radiohumeral plica being the most commonly symptomatic [268] (Fig. 14). No studies directly compare cMRI to dMRA for evaluating elbow plica. Several studies report evaluation of plica with conventional arthrography [270–274], some in combination with dMRA, while others used cMRI [275, 276]. Additional studies suggest evaluation is best in the presence of a joint effusion or with arthrography [275, 277, 278].

*Recommendation: cMRI recommended (consensus recommendation, supermajority (11/12)).*

##### Pediatric elbow

Beyond the evaluation of osteochondral lesions, as discussed previously, the use of dMRA of the elbow in pediatrics is limited. Post-operative dMRA has been used to assess fixation alignment following lateral epicondylar fractures in children; however, a recent study suggests that this has not changed management [279, 280]. Further study is warranted; however, preoperative US may best assess articular congruence and aid operative decision-making [280].

Aside from trauma, pediatric elbow evaluation is frequently performed for overuse injuries. Elbow overuse injuries are commonly encountered in young athletes competing in gymnastics and overhead throwing sports, particularly baseball [281]. Conventional MRI provides adequate evaluation of these injuries in most cases and imaging signs indicating instability have been described [282, 283]. Direct MR arthrography can be helpful when cMRI is inconclusive.

*Recommendation: cMRI recommended (consensus recommendation, unanimous).*

### Wrist

#### Technical considerations

A single compartment injection into the radiocarpal joint is the preferred method for most indications [28]. An additional injection into the distal radioulnar joint (DRUJ) may be considered in setting of ulnar-sided pain [284]. A tricompartmental joint injection, including a mid-carpal joint injection, has been suggested by some authors in cases of chronic wrist pain with unclear origin [79]. However, the communication of injected contrast material from the radiocarpal joint to the midcarpal joint is a helpful imaging feature of intrinsic ligament tear that is obscured by injection of the midcarpal joint space. We do not feel that a midcarpal compartment injection is necessary when performing dMRA of the wrist.

The procedure may be performed with the patient seated or prone with the hand in an outstretched position. Although rare, vasovagal episodes could decrease using the prone position. For radiocarpal injection, the needle should be directed toward the radio-scaphoid space, closer to the proximal edge of the scaphoid and with the wrist positioned in ulnar deviation and flexion [285]. Some authors recommend choosing an injection site away from the site of symptoms. Thus, in cases of radial-sided pain, an ulnar-sided injection with the needle directed to the pisiform-triquetrum recess at the proximal edge of the triquetrum may be considered [79]. However, if positioned properly, a radial-sided injection may avoid proximity to the scapholunate ligament and allow adequate diagnostic evaluation. If additional DRUJ injection is to be considered, the needle should be directed to the head of the ulna along its radial margin, keeping in mind that this joint surrounds the head of the ulna.

A total volume of 3–4 mL is recommended for radiocarpal joint injection, with another 3 mL considered if there is communication with the midcarpal joint. An additional 1 mL of contrast may be added with DRUJ contrast leakage [79]. Isolated DRUJ injection volume without leakage should be limited to 1–2 mL [79].

Additional “stress” fluoroscopic views may then be obtained after removal of the needle and subsequent wrist exercise (i.e., repeated flexion-extension and “motion” of the wrist) with radial and ulnar deviation as well as anteroposterior and lateral projections [286, 287]. Provocative maneuvers, such as clenched-fist views, are helpful in differentiating gaping tears with spontaneous leak of contrast from small perforations with a valve function. Wrist traction during MR scanning may improve tear detection involving the intrinsic ligaments and triangular fibrocartilage complex (TFCC) [288].

#### Clinical indications

Individual investigators and expert panels have proposed guidelines for the appropriate use of dMRA of the wrist [79, 284, 289, 290]. Selection of patients for wrist dMRA is best dictated by the clinical indication.

##### Scapholunate and lunotriquetral interosseous ligaments

**Fig. 15:**
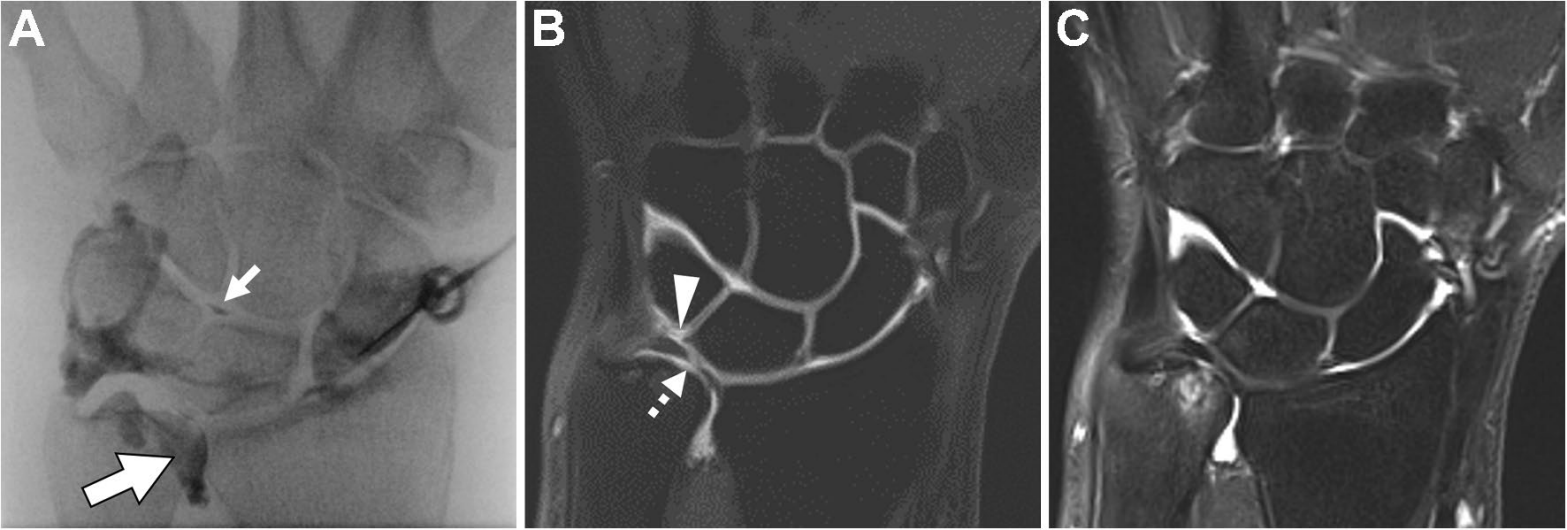
Suspected internal derangement of the wrist. Fifty-five-year-old man with wrist pain. **A** Spot image of the wrist after radiocarpal joint injection shows flow of contrast into the midcarpal (small arrow) and distal radioulnar (big arrow) joints. Coronal T1-weighted 1.5 T MR arthrogram image (**B**) shows a tear of the lunotriquetral interosseous ligament (arrowhead) and triangular fibrocartilage (dashed arrow), which were not as clearly shown on the coronal T2-weighted fat-suppressed image (**C**)

Both cMRI and dMRA detect and characterize intrinsic ligament injuries [291–293]. Several studies assess the relative accuracy of 1.5 T and 3 T cMRI and dMRA in diagnosing scapholunate interosseous ligament (SLIL) injuries and to better define the appropriate role of advanced imaging [5, 294–296]. A meta-analysis of 24 studies indicated pooled sensitivity and specificity of dMRA of 82.1% and 92.8%, respectively in the detection of SLIL injury. In the same analysis, 3 T cMRI pooled sensitivity and specificity were 75.7% and 97.1%, respectively, while 1.5 T cMRI pooled sensitivity and specificity were 45.7% and 80.5%, respectively [292]. It is likely that some component of the superior diagnostic accuracy reported with 3 T systems relative to 1.5T systems is related to improved coil technology and scanning techniques that have developed in parallel with more widespread adoption of high field strength scanners. Fewer studies have evaluated the performance of cMRI and dMRA in evaluation of the lunotriquetral interosseous ligament (LTIL) injuries [5, 294]. Despite the minimally invasive nature of the examination, given its superior sensitivity, dMRA is recommended for evaluation of the SLIL and LTIL in the setting of suspected injury and instability if imaging at 1.5 T (Fig. 15). When imaging at 3 T, cMRI is a reasonable alternative to dMRA.

*Recommendation: dMRA or cMRI recommended* when imaging at 3 T. *dMRA recommended* when scanning at a field strength of 1.5 T or lower **(***consensus recommendation, unanimous***)**.

##### TFCC

**Fig. 16:**
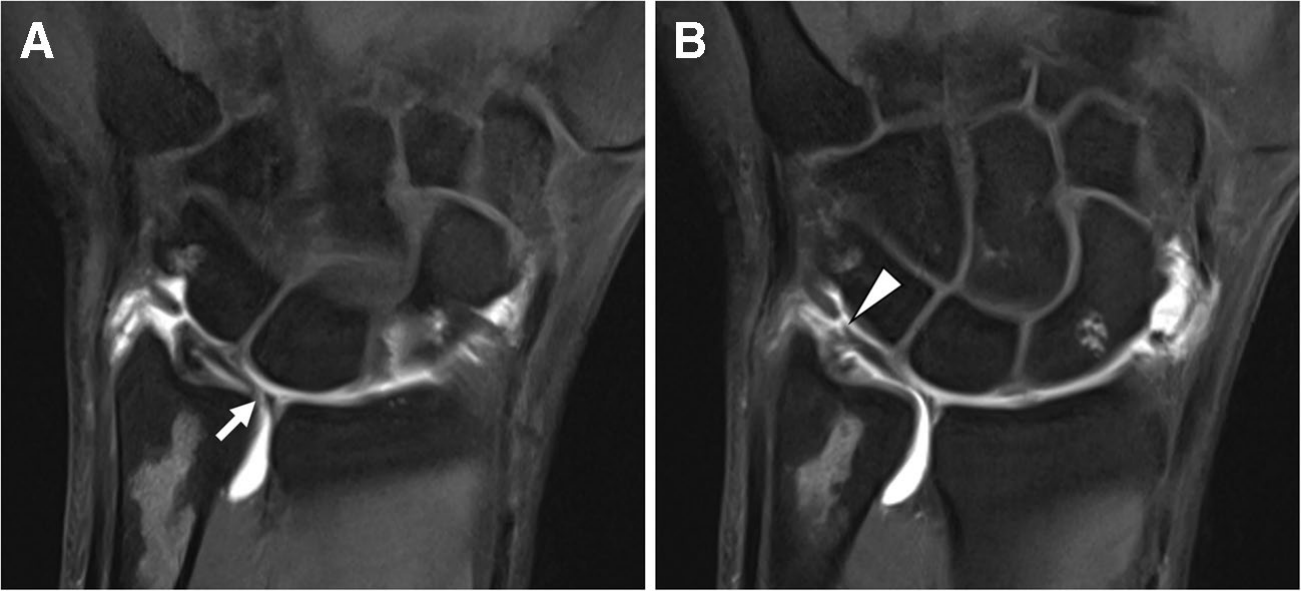
Triangular fibrocartilage complex (TFCC) injury. **A**, **B** Coronal T1-weighted 3T MR arthrogram images show communication between the radiocarpal and distal radioulnar joints through a tear of the TFCC involving both the central disc (arrow) and ulnar attachments (arrowhead). Findings were confirmed at arthroscopy and an open foveal repair was performed

Imaging evaluation of the triangular fibrocartilage complex is challenging given the complexity of the structure, variability of appearance and common presence of asymptomatic abnormalities [297–299]. Some studies have shown a lack of diagnostic value associated with cMRI imaging of the TFCC and inferiority to wrist arthroscopy [300, 301]. Despite this, 1.5 T cMRI and 3 T cMRI as well as dMRA have been utilized in the evaluation of the TFCC with some evidence that diagnostic accuracy is improved with increased field strength [302–307]. A meta-analysis reported pooled sensitivity and specificity of dMRA of 78% and 85%, respectively, for the diagnosis of TFCC injury. In the same analysis, cMRI pooled sensitivity and specificity were 76% and 82% [308]. In several individual studies however, dMRA has been shown to confer additional advantages in diagnostic accuracy, though it remains debated whether this benefit outweighs the cost and small risks associated with the minimally invasive procedure [5, 295, 309–314]. It has also been shown that the inclusion of an injection at the distal radioulnar joint may be of benefit in the diagnosis of peripheral TFCC tears [284, 315]. While conflicting literature exists regarding the diagnostic value of cMRI and dMRA, there is sufficient evidence to support the use of dMRA in the diagnosis of ulnar-sided wrist pain when there is clinical suspicion of injury to the TFCC (Fig. 16).

*Recommendation: dMRA or cMRI recommended* when imaging at 3 T. *dMRA recommended* when scanning at a field strength of 1.5 T or lower **(***consensus recommendation, unanimous***)**.

##### Postoperative imaging

**Fig. 17:**
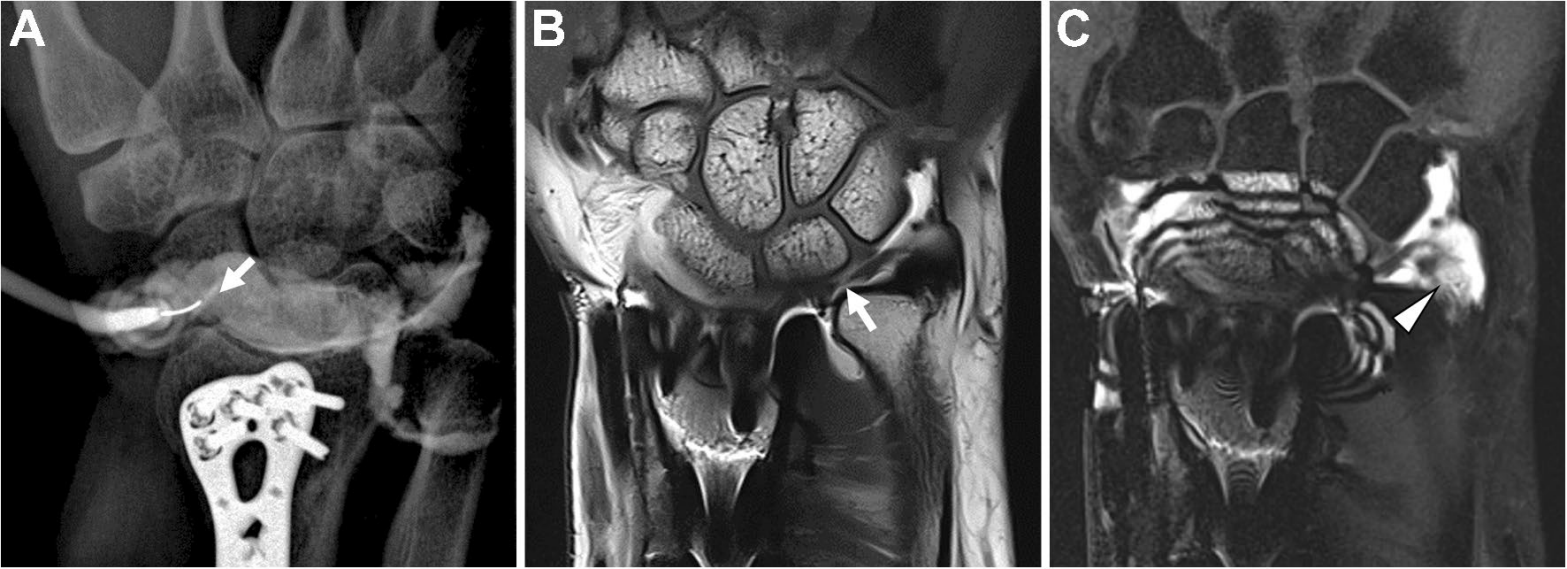
Postoperative wrist imaging. Thirty-eight-year-old man with wrist pain following radius fracture and surgery. **A** Fluoroscopic-guided direct arthrogram image shows flow of contrast from the radiocarpal joint into the distal radioulnar joint. Coronal T1-weighted 1.5 T MR arthrogram images without (**B**) and with (**C**) fat-suppression show the triangular fibrocartilage complex tear involving the central disc (arrow) and ulnar attachments (arrowhead), but the scapholunate interosseous ligament is obscured by artifact. The integrity of the scapholunate and lunotriquetral interosseous ligaments can be assumed by the lack of contrast extension into the midcarpal compartment

Postoperative imaging is challenging due to altered anatomy and variable signal intensities of involved tissue structures. Direct MR arthrography is useful in demonstrating abnormal flow of injectate between wrist compartments or through torn ligament structures, particularly when they demonstrate heterogeneous signal characteristics [79]. For this reason, dMRA may be favored for the same pre-operative indications, while high-resolution 3 T cMRI serves as an acceptable alternative. Of note, the direct fluoroscopic visualization of intrinsic ligament or TFCC tears may prove particularly useful in cases where extensive susceptibility artifact limits MR image quality (Fig. 17).

*Recommendation: dMRA or cMRI recommended* when imaging at 3 T. *dMRA recommended* when scanning at a field strength of 1.5 T or lower **(***consensus recommendation, unanimous***)**.

## Hip

### Technical considerations

For dMRA of the hip, the patient should lie on the table in a supine position [19]. The limb is positioned in 10–15° of internal rotation; a sandbag or other weight applied to the outside of the foot may assist in holding the position [316]. The typical anatomic target for intra-articular access is the superolateral aspect of the femoral head-neck junction [12, 19]. This will avoid the femoral neurovascular bundle, the iliopsoas tendon, and the zona orbicularis [12, 19]. The simplest approach is a direct vertical needle path to reach this point [19]. Alternatively, an oblique vertical approach may also be chosen, utilizing a more lateral start point on the skin surface to avoid the lateral femoral cutaneous nerve [19]. The midportion of the femoral neck should be avoided because the zona orbicularis can be challenging to penetrate [12]. An injected volume of 10–12 mL typically provides adequate distention of the hip joint [19]. Volumes closer to 15 mL may result in overdistention, which can cause leakage of contrast from the joint puncture site [19].

Large field of view imaging (e.g., 30–40 cm) is inferior for the detection of labral and chondral abnormalities, but may be added to assess for extra-articular pathology [119]. With regards to radial imaging on dMRA exams, there are mixed results in the literature. In a cadaveric study evaluating radial imaging compared with conventional oblique coronal and oblique axial planes, radial imaging increased sensitivity and accuracy of labral tear detection from 60 to 75% and 70 to 85%, respectively (both techniques were notably 100% specific for the detection of labral tears) [317]. In another study that included 54 dMRA exams of the hip, radial imaging did not demonstrate any labral tears that were not identified on standard imaging planes [318]. Radial imaging can be helpful to evaluate the femoral head-neck junction and characterize regions of cam morphology, however, that may not be as evident on standard imaging planes [119].

*Recommendation: Radial imaging may be recommended (consensus recommendation, unanimous).*

#### Clinical indications

##### Labrum

**Fig. 18:**
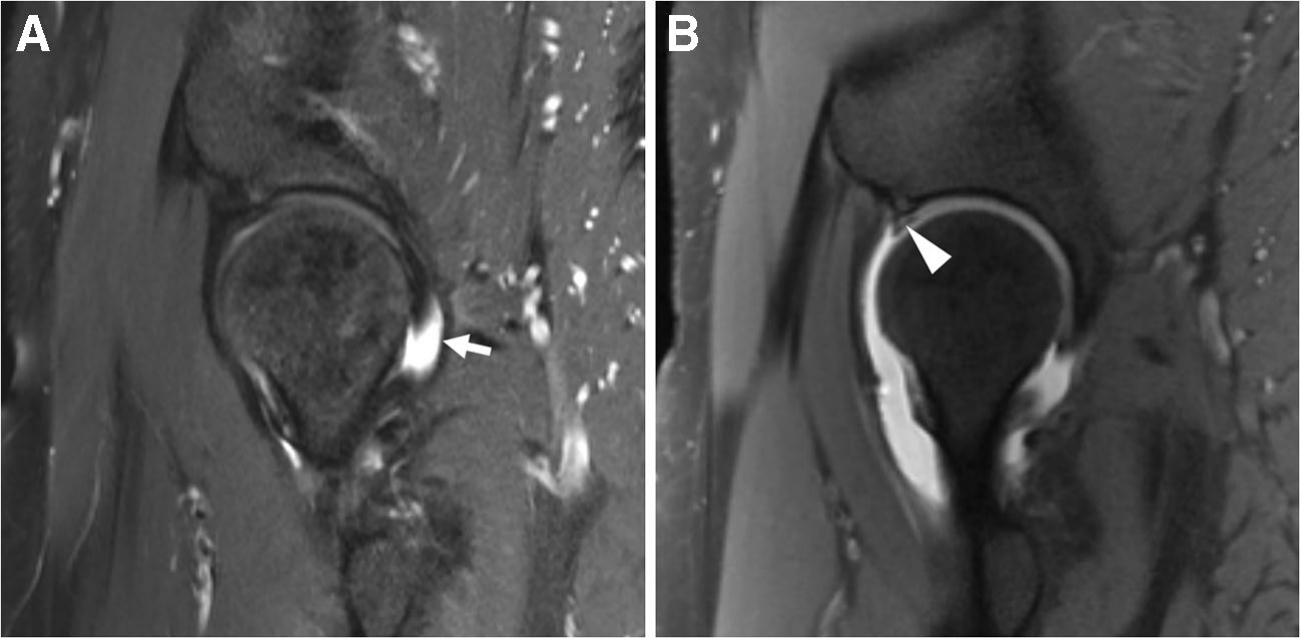
Labral tear. Forty-nine-year-old woman with hip pain. **A** Sagittal T2-weighted fat-suppressed conventional 3 T MR image shows a small, native joint effusion (arrow), but a labral tear is not clearly demonstrated. **B** Sagittal T1-weighted fat-suppressed 3 T MR arthrogram image shows contrast extending into the substance of the anterosuperior labrum (arrowhead), consistent with a tear which was subsequently confirmed at arthroscopy

Historically, dMRA was felt to be superior to cMRI for the detection of acetabular labral tears [120, 319] (Fig. 18). However, many of the original studies were performed at 1.5 T and with lower spatial resolution compared with typical 3 T protocols [119]. As such, the need for arthrography to diagnose acetabular labral tears has become more controversial in recent years [122, 320]. An updated meta-analysis showed a pooled sensitivity and specificity of 89% and 69% for dMRA, compared to 80% and 77% for cMRI [321]. In one study, when considering 3 T cMRI alone, sensitivity was similar to dMRA (87%), and specificity was superior (77%) [321]. The literature remains inconclusive regarding 3 T cMRI versus 1.5 T dMRA, partly due to the lack of properly designed studies. However, based on publications to date, it appears that high-quality 3 T cMRI of the hip is at least equivalent to 1.5 T dMRA for the detection of labral tears. Comparisons of cMRI and dMRA of the hip at 3 T have been sparse, and there are conflicting results regarding whether 3 T cMRI is equivalent or inferior to 3 T dMRA in detecting labral tears [122, 320].

*Recommendation: dMRA or cMRI recommended* when imaging at 3 T. *dMRA recommended* when scanning at a field strength of 1.5 T or lower **(***consensus recommendation, unanimous***)**.

##### Ligamentum teres

**Fig. 19:**
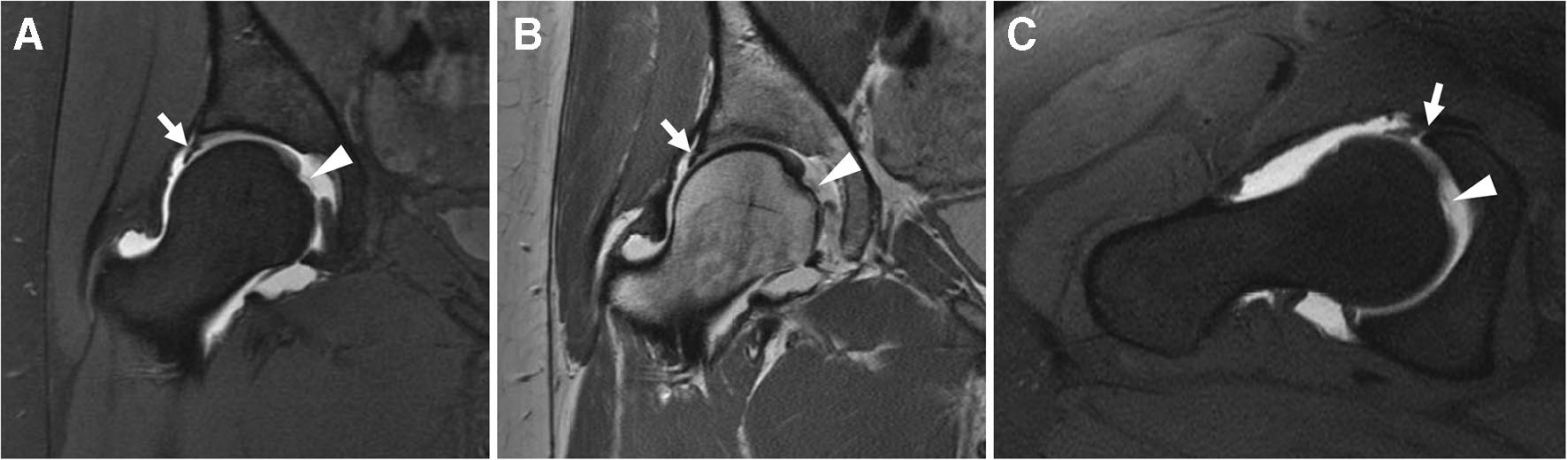
Ligamentum teres tear. Twenty-eight-year-old ex-ballerina with chronic hip pain. Coronal T1-weighted fat-suppressed (**A**), coronal intermediate-weighted (**B**), and axial oblique T1-weighted fat-suppressed MR arthrogram images show chronic tearing and resorption of the ligamentum teres (arrowheads) as well as a torn labrum (arrows)

Initially overlooked, injuries to the ligamentum teres are now recognized as potential generators of pain and instability [322, 323]. These injuries are also relatively common, accounting for up to 15% of sports-related hip injuries [323]. The clinical diagnosis of ligamentum teres injuries is challenging, making imaging diagnosis valuable [322] (Fig. 19).

Direct MRA has shown superior accuracy for the detection of ligamentum teres abnormality compared to cMRI; a meta-analysis of 8 studies reported sensitivity and specificity of 82% and 89% for dMRA, compared to 65% and 87% for all MR examinations (a separate diagnostic performance could not be calculated for cMRI from 2 studies) [322].

*Recommendation: dMRA or cMRI recommended (consensus recommendation, unanimous).*

##### Instability

Hip instability has a wide range of presentations, from microinstability to complete dislocation [324]. Microinstability, defined as abnormal hip motion without frank subluxation or dislocation, has recently been recognized as a cause of hip pain in young adults [324]. It is important that this diagnosis be made preoperatively, as it may exist in conjunction with impingement and typical surgical treatments may worsen the instability [324].

Preliminary studies have begun to address the question of how best to assess microinstability on imaging. The cliff sign on radiographs is said to be highly specific for hip micro-instability, up to 100% sensitive and specific in women under 32 years [325]. One study suggested utilizing dMRA to visualize a crescent shaped pooling of contrast in the posteroinferior joint space, and the crescent sign has recently been proposed as being relatively specific for the diagnosis of hip instability [243]. Another study of dMRA found a thinner anterior capsule (2.5 mm) and wider anterior recess (5.8 mm) in patients with capsular laxity at arthroscopy [326]. Finally, in a small series of patients with iatrogenic instability due to capsular defect following hip arthroscopy, 78% of patients had a capsular defect visible on dMRA [327].

A recent consensus on intra-operative criteria for the diagnosis of hip micro-instability has been published [328]. However, at MR imaging, the diagnostic criteria are not clear. There is some evidence that anterior labral tears, ligamentum teres tears, and dysplastic morphology may be associated with microinstability in the appropriate clinical scenario [324]. While dMRA may be helpful to assess these features, a recent systematic review determined that there is not yet sufficient evidence to support any particular imaging features as being diagnostic for microinstability [324].

*Recommendation: cMRI recommended (consensus recommendation, unanimous).*

##### Postoperative imaging

**Fig. 20:**
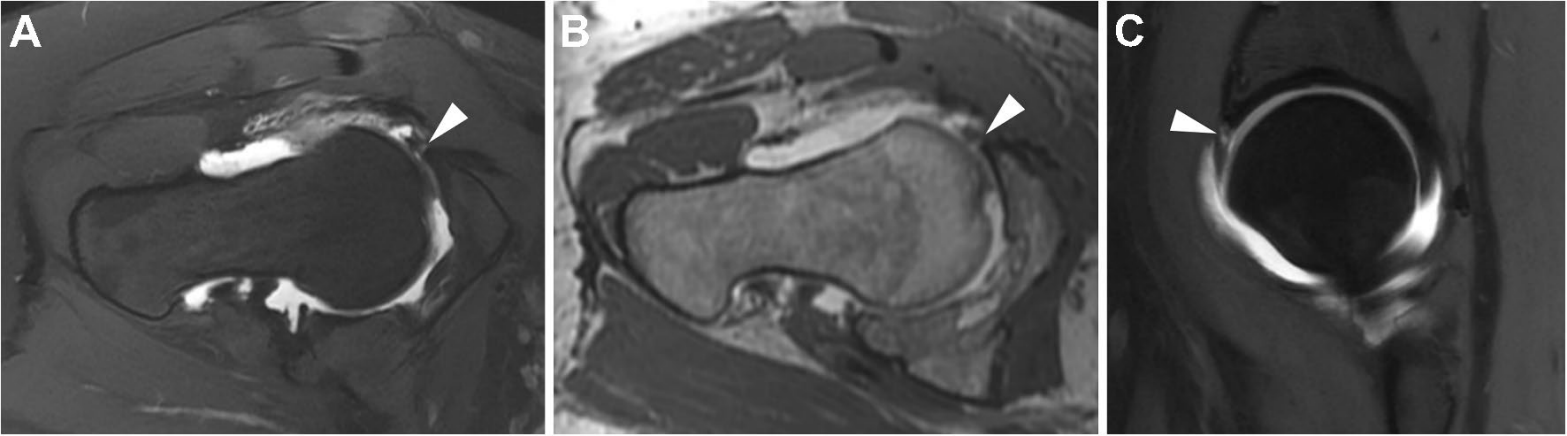
Postoperative hip imaging. Twenty-one-year-old woman with prior resections of cam and pincer lesions as well as a periacetabular osteotomy, presenting with hip pain. Axial oblique T2 fat-suppressed (**A**), radial T2-weighted (**B**), and sagittal T1-weighted fat-suppressed MR arthrogram images show a surgically confirmed recurrent tear of the labrum extending from 10 o’clock to 2 o’clock (arrowheads)

Following hip arthroscopy, residual, recurrent, or new symptoms may be related to the original lesion(s), new lesions, or the surgical procedure itself. The role of imaging in these scenarios is evolving as is the optimal method in which to image a post-operative hip. Common causes of post-operative symptoms include incomplete resection of cam morphology, labral tears, cartilage lesions, adhesions, capsular defects with or without instability, or, very rarely, osteonecrosis or fractures [329–332] (Fig. 20).

Although high resolution 3 T cMRI appears to be adequate for the evaluation of the labrum, significant cartilage lesions and the rare case of fracture or osteonecrosis, dMRA may be needed to assess intra-articular adhesions and capsular defects. The performance of cMRI vs dMRA for the detection of residual or recurrent labral tears after labral repair is unknown. However, caution is advised as intra-articular abnormalities of the labrum, cartilage, adhesions, and capsular defects have been reported to occur with the same prevalence in symptomatic and asymptomatic patients after hip arthroscopy [333].

*Recommendation: dMRA recommended (consensus recommendation, unanimous).*

### Knee

#### Technical considerations

In a study surveying musculoskeletal radiologist preference for knee arthrographic approach, 64% percent preferred a lateral patellofemoral approach, 25% the medial patellofemoral approach, and 11% the arthroscopic approach. 9% performed knee arthrograms using palpation alone, without imaging guidance [334], though this may not be advisable for dMRA. A variation on the anterior paramedian approach is the anterolateral approach, where the needle is directed towards the lateral trochlear cartilage instead of the intercondylar notch [335]. One study found a small but statistically significant reduction in both absolute and relative pain when employing the anterolateral approach [335].

The volume of injectate used for dMRA varies in the literature, ranging from 20 to 50 ml, with some authors titrating to perceived resistance to the injection while monitoring distention with fluoroscopy [61, 64, 69, 75, 77, 336, 337]. One study found that increasing from 20 ml of injectate to 40 ml of injectate and suprapatellar compression with an elastic bandage did not significantly increase the presence of gadolinium signal intensity in meniscal tears [338]. Direct MRA with axial traction has been shown to increase the contrast material between the femorotibial articular surfaces [336], but studies showing an improvement in diagnostic accuracy using either axial traction or suprapatellar compression with dMRA are absent. For dMRA, the authors suggest a minimum of 20 ml of injectate, with volumes between 30 and 40 ml recommended.

There is variation in the approach towards joint activity following arthrography for dMRA. In some studies, patients complete 5 min of repeated knee flexion and extension prior to MR imaging [64, 339]; others have patients walk from the fluoroscopy suite to the MRI scanner [338]. The theoretical benefit of exercise is to distribute the injectate throughout the joint and promote the imbibition of fluid/gadolinium into meniscal tears or chondral abnormalities [61, 340]. Since the risk of extraarticular contrast leakage of injectate following knee arthrography is considered low, the authors recommend exercising the knee prior to MR imaging. The incorporation of weight-bearing by having patients walk a short distance seems reasonable for this purpose, although care should be used if patients exhibit vasovagal symptoms or signs.

With the widespread use of the electronic medical record and picture archiving and communication systems (PACS) and the ability to electronically store and distribute medical images, when evaluating the post-operative meniscus, every effort should be made to obtain both the pre-operative MRI scan and the surgical report to aid in MRI interpretation [341]. The authorship panel also recommends including FSE/TSE T2-weighted imaging (with or without fat-suppression) in their conventional post-operative knee MRI protocol in the sagittal and/or coronal plane.

#### Clinical indications

##### Postoperative meniscus

**Fig. 21:**
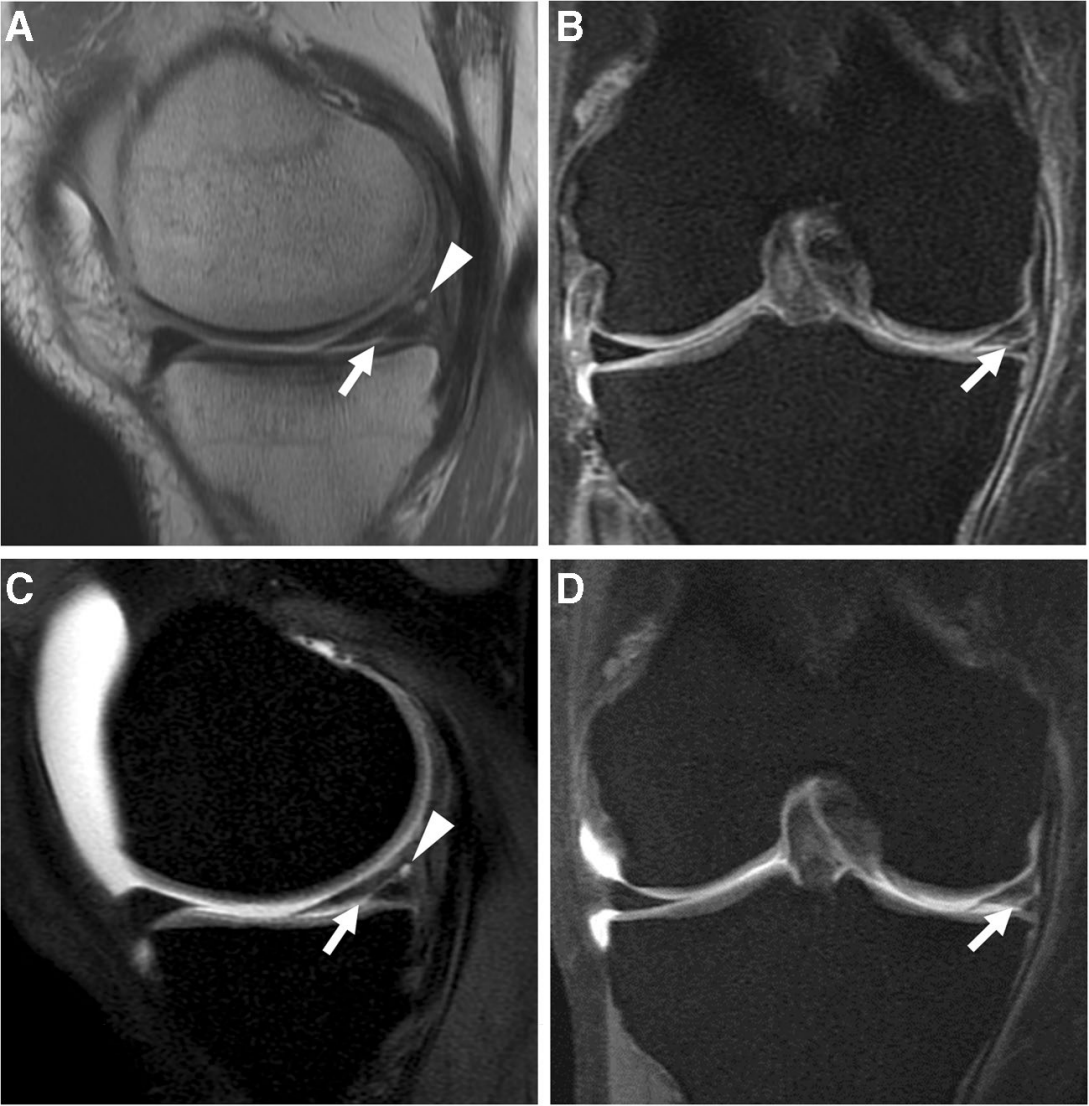
Recurrent meniscal tear. Fifty-one-year-old man with history of meniscal surgery and recurrent symptoms. Sagittal intermediate-weighted (**A**) and coronal intermediate-weighted fat-suppressed (**B**) conventional MR images show evidence of partial medial meniscectomy with linear increased signal extending to the inferior surface (arrows) and small posterior parameniscal cyst (arrowhead), consistent with a recurrent tear. Sagittal (**C**) and coronal (**D**) T1-weighted fat-suppressed MR arthrogram images demonstrate dilute gadolinium extending through the tear (arrows) and filling the parameniscal cyst (arrowhead)

In the presence of partial meniscectomy involving less than 25% of the meniscus, studies support the use cMRI for the diagnosis of a recurrent meniscal tear (with up to 100% accuracy) [69, 337, 339, 340, 342, 343]. However, in the presence of partial meniscectomy involving greater than 25% of the meniscus, multiple studies have found dMRA to be superior to cMRI for the diagnosis of recurrent meniscal tears [64, 339, 343], although one prospective study reported no statistically significant difference in diagnostic accuracy (80% for cMRI versus 85% for dMRA, *p* > .54) [69] (Fig. 21). Of note, only one of these studies comparing cMRI and dMRA at 3T was published in the past decade [64], with the others published in 1993, 2002, and 2003 [69, 339, 343].

In a recent retrospective cMRI study to evaluate for a recurrent meniscal tear following partial meniscectomy in 140 patients (148 menisci) with comparison to second-look arthroscopy, the authors found the absence of a T2 signal line extending to the meniscus articular surface had a negative predictive value of 100% for recurrent meniscal tear, whereas an intermediate to high T2 signal line extending to the articular surface had sensitivity of 40.4%, specificity of 95.8% and positive predictive value of 90.9% for recurrent tear [344]. Overall, the most useful characteristic for the detection of a torn postoperative meniscus was a change in the meniscus signal intensity pattern compared to the baseline MRI, with a sensitivity of 85.7%, specificity of 98.2%, and positive predictive value of 99.4% [344]. The authors did not stratify patients based on the percentage of meniscal resection.

Following meniscal repair, studies comparing cMRI and dMRA are limited by small sample sizes and have showed mixed results [68, 69, 339, 343]. In some studies, results for cMRI and dMRA were identical, with both showing high accuracy (89–100%) [69, 343]. In contrast, in another study with 16 patients following meniscal repair, cMRI was unable to differentiate between a healed repair from a residual tear in all cases, whereas dMRA was able to make the correct diagnosis [339]. The largest series of dMRA following meniscal repair included 24 symptomatic patients and sensitivity, specificity and accuracy was reported at 80%, 100%, and 84.6% respectively [345].

*Recommendation: cMRI recommended.* However, dMRA may be beneficial when cMRI fails to identify an etiology for the patient’s symptoms, when there is a high clinical suspicion for a recurrent or residual meniscal tear, or when details of the prior surgical procedure are lacking and a pre-operative cMRI is not available for direct comparison **(***consensus recommendation, majority (9/12)***)**.

##### Plica

One study found that dMRA could be used to detect 17 of 19 (89%) mediopatellar plicae whereas only 3 of 11 (27%) could be detected by cMRI [346]. A systematic review and meta-analysis reported a pooled sensitivity of 77% and pooled specificity of 58% for cMRI in the detection of medial patella plica syndrome [347]. Other authors have also shown that detection of suprapatellar, infrapatellar, and lateral patellar plicae is possible using dMRA, without head-to-head comparisons with cMRI [61, 348].

*Recommendation: cMRI recommended (consensus recommendation, unanimous).*

### Ankle/foot

#### Technical considerations

Ankle arthrography is performed under fluoroscopic guidance with either the anteromedial or lateral mortise approach. For the former, an anteroposterior view of the ankle is obtained to mark the point of access medial to the tibialis anterior tendon. Care should be taken to avoid the tibialis anterior artery, which can be localized by palpation. The patient is then turned to the lateral decubitus position while the tibiotalar joint is accessed using fluoroscopic guidance in the lateral plane [60, 349]. In the latter, a mortise view is obtained and the needle is directed into the fibulotalar space [350]. With dMRA of the talocrural joint, it should be noted that intra-articular solutions extend into the flexor hallucis and flexor digitorum tendon sheaths as well as the subtalar joint in 25% of the cases [60]. A volume of 4–8 mL of injectate is recommended for dMRA, with volumes in the higher range when normal regional joint or tendon sheath communications are observed [60, 351].

#### Clinical indications

##### Ligament injuries

Multiple studies suggest that dMRA may improve the delineation of the ligaments as capsular distention with contrast assists in separating the ligaments from the underlying structures [349, 352, 353]. A meta-analysis has shown that dMRA has pooled sensitivity and specificity of 100% for chronic ATFL injuries [354]. Extra-articular leakage of contrast may be observed as a sign of ligament injury. Direct MRA can demonstrate contrast communication between the ankle joint and the peroneal tendon sheath for example, indicating a full thickness calcaneofibular ligament tear. However, cMRI has also been shown to be highly accurate for diagnosis of ATFL (accuracy = 97%) [355], deep deltoid ligament (sensitivity = 82–96%; specificity = 98–100%) [356, 357], and syndesmotic ligament complex injuries (pooled sensitivity and specificity of 93% and 87%) [358].

*Recommendation: cMRI recommended (consensus recommendation, unanimous).*

##### Ankle impingement syndromes

Anterolateral impingement (ALI) occurs in 2–3% of patients with ankle sprains [359–361], typically in young athletic males [359, 362, 363]. A single prospective study reported a sensitivity of 96% and specificity of 100% of dMRA for assessment of anterolateral soft tissues in 32 patients, with higher accuracy (100%) than cMRI in 13 patients with ALI [360]. A highly specific, but insensitive dMRA finding was absence of a normal fluid-filled recess between the anterior fibula and the anterolateral soft tissues. A cMRI study found that a substantial joint effusion was required to accurately assess the anterolateral recess, supporting the contention that articular distention with fluid allows more precise diagnosis of ALI [364]. Conventional MRI demonstrates high performance for findings associated with ALI, with sensitivities as high as 83% and specificities of 75–100% [365, 366]. A single CT arthrography study in 41 patients with ALI calculated a similar sensitivity to dMRA of 97%, but a lower specificity of 71% [367]. It should be noted that 11 of the 19 control patients in one study had scarring or synovitis in their anterolateral recess on both dMRA and arthroscopy. This reaffirms the fact that ALI is a clinical diagnosis, and the presence of a potential impingement lesion alone is insufficient to make the diagnosis [360, 368].

Anteromedial impingement (AMI) may develop secondary to soft tissue injury, making cMRI and dMRA more useful than radiography and CT [362, 369]. In addition to demonstrating ligamentous abnormalities, dMRA increases the conspicuity of focal synovitis, fibrosis, and scar formation in the anteromedial recess [60, 370]. A single prospective study of dMRA in two patients with AMI concluded that dMRA improves the conspicuity of soft tissue pathology related to AMI compared with cMRI [371]. This conclusion is disputed by others [368, 370]. Overall, available data is not sufficient due to the rarity of this condition.

*Recommendation: cMRI recommended (consensus recommendation, unanimous).*

##### Metatarsalgia

The use of dMRA has also been described in the metatarsophalangeal joints (MTPJs). Cadaveric studies have shown improved visualization of articular structures in the first and lesser MTPJs using dMRA compared with cMRI at 1.5 T [372–374]. Clinical studies have also suggested utility of dMRA for demonstrating capsular and plantar plate tears in the lesser MTPJs [375–377], though there is a paucity of head-to-head studies in comparison with cMRI. As there is often a joint effusion and/or synovitis present which provides most of the diagnostic benefits of dMRA without the invasive procedure [378, 379], many authors emphasize high-quality cMRI exams with dedicated surface coils rather than dMRA [378–381]. A recent meta-analysis calculated 89% pooled sensitivity and 83% pooled specificity of MRI (dMRA plus cMRI) for plantar plate tears in comparison to ultrasound with a 95% pooled sensitivity and 52% pooled specificity [382]. Despite lower specificity, ultrasound was recommended as a less expensive and preferred screening test. Overall, dMRA of the MTPJs is not commonly performed and likely unnecessary for the patient’s initial MRI exam.

*Recommendation: cMRI recommended (consensus recommendation, unanimous).*

## Tumor arthrography

Direct MR arthrography can be utilized for tumor applications. Conventional MRI often provides adequate tumor detail, including any joint association. In select cases, pathology adjacent to joints can pose a diagnostic dilemma for surgical planning [383]. Direct MRA definitively assesses intraarticular location or extension [384–388]. For example, subchondral pathology may be large or not immediately subchondral and dMRA can assess for potential joint communication [389]. With intraneural ganglion cysts and adventitial cystic disease, credible evidence supports their connection to an adjacent joint [390, 391], which typically is detected by conventional MRI [392]. In select cases, dMRA can establish a joint connection [61, 78].

*Recommendation: cMRI recommended (consensus recommendation, unanimous).*

## Controversies and gaps in the literature

When access to 3 T imaging is available, the additional diagnostic yield of dMRA is less pronounced. This trend will likely continue as hardware and software technology continues to improve. The majority of studies including dMRA are retrospective in design and include small sample sizes. Studies directly comparing 3 T cMRI, 1.5 T cMRI, 3 T dMRA, and 1.5 T dMRA groups are few, and better controlled or randomized studies would help define best imaging approaches to diagnoses with equipoise. While systematic reviews and meta-analyses provide useful information, if a study does not consider the impact of dated equipment and software, the reported test accuracy estimates may not apply to a user with the latest technology in their practice [393].

It is important to keep in mind that statistically significant, but marginal improvements in diagnostic performance in a research study may not necessarily equate to high clinical value. Considerations of cost-effectiveness are beyond the scope of this white paper but are extremely important in real-world settings. The cost-effectiveness of dMRA has been studied by some authors, most notably in the upper extremities [394–396]. One study found that, for SLAP tears, dMRA is only cost-effective when 3 T is unavailable [396]. More studies assessing value and cost-effectiveness are necessary, including comparison with lower-cost modalities such as US.

Other notable gaps in the literature include direct comparisons between dMRA and cMRI in the assessment of chondral and osteochondral abnormalities (particularly in the shoulder, elbow, wrist, and ankle) as well as post-surgical conditions such as prior rotator cuff repair and following meniscal root repair.

## Conclusion

Although dMRA has been previously used for a wide variety of clinical indications, the authorship panel recommends more selective application of this minimally invasive procedure (Table 3). When joint imaging is performed on 3T scanners using modern coils and software technology, the incremental benefit of dMRA is reduced. For some joints, the current dMRA and cMRI literature supports this premise, as reflected in the panel’s differing joint specific recommendations for 3 T compared to 1.5 T (or lower) for imaging the wrist and hip. High quality comparative studies are required to determine if this principle can be applied to intra-articular joint pathology in other joint-specific or pathology-specific MR imaging indications. At present, direct MR arthrography remains an important procedure in the armamentarium of the musculoskeletal radiologist and is especially valuable when cMRI is indeterminant or results are discrepant with clinical evaluation.

**Table 3:** Summary of recommendations for the utilization of direct MR arthrography as the initial MRI evaluation

Indication	Recommendation	Comments
Pathology specific indications		
Chondral and osteochondral abnormalities	B	
Post-operative evaluation of chondral and osteochondral abnormalities	B	
Intra-articular Bodies	C	
Joint specific indications		
Shoulder		
Provocative maneuvers		*Recommendation:* Provocative maneuvers, including ABER, are not recommended in routine practice.
Instability	B	*Recommendation:* dMRA or cMRI recommended. Direct MRA has a compelling role in the assessment of younger individuals with suspected instability, when subtle labroligamentous abnormalities may have profound influences on shoulder function, management, and prognosis.
Humeral avulsion of the glenohumeral ligament (HAGL)	B	
Thrower’s shoulder	B	
Recurrent instability and post-operative labrum	A	
Rotator cuff tear and retear	C	
Long head biceps tendon (LHBT) and pulley lesions	C	
Elbow		
Posterolateral rotatory instability (PLRI)	C	
Valgus injury/thrower’s elbow	B	
Plica	C	
Pediatric elbow	C	
Wrist		
Scapholunate and lunotriquetral interosseous ligaments	B (3 T) A (≤ 1.5 T)	*Recommendation:* dMRA or cMRI recommended when imaging at 3T. dMRA recommended when scanning at a field strength of 1.5T or lower.
TFCC	B (3 T) A (≤ 1.5 T)	*Recommendation:* dMRA or cMRI recommended when imaging at 3T. dMRA recommended when scanning at a field strength of 1.5T or lower.
Postoperative imaging	B (3 T) A (≤ 1.5 T)	*Recommendation*: dMRA or cMRI recommended when imaging at 3T. dMRA recommended when scanning at a field strength of 1.5T or lower.
Hip		
Radial imaging		*Recommendation:* Radial imaging may be recommended.
Acetabular labrum	B (3 T) A (≤ 1.5 T)	*Recommendation*: dMRA or cMRI recommended when imaging at 3T. dMRA recommended when scanning at a field strength of 1.5T or lower.
Ligamentum teres	B	
Instability/microinstability	C	
Postoperative hip	A	
Knee		
Postoperative meniscus	C	The authorship panel recommends including FSE/TSE T2-weighted imaging (with or without fat-suppression) in their conventional post-operative knee MRI protocol.
Plica	C	
Ankle/foot		
Ligament injuries	C	
Ankle impingement syndromes	C	
Metatarsalgia	C	
Tumor arthrography	C	Conventional MRI often provides adequate tumor detail, including any joint association. With intraneural ganglion cysts and adventitial cystic disease, evidence supports their connection to an adjacent joint, which typically is detected by conventional MRI.

A practitioner may appropriately choose to supersede a panel recommendation based on their expertise and experience in a specific clinical scenario and in consultation with the referring physician. Panel recommendations: (A) dMRA recommended; (B) dMRA or cMRI recommended; (C) cMRI recommended
