# Correction to: SSR white paper: guidelines for utilization and performance of direct MR arthrography

**DOI:** 10.1007/s00256-023-04452-y

**Published:** 2023-09-11

**Authors:** Eric Y. Chang, Jenny T. Bencardino, Cristy N. French, Jan Fritz, Chris J. Hanrahan, Zaid Jibri, Ara Kassarjian, Kambiz Motamedi, Michael D. Ringler, Colin D. Strickland, Christin A. Tiegs‑Heiden, Richard E. A. Walker

**Affiliations:** 1 Radiology Service, VA San Diego Healthcare System, San Diego, CA USA; 2 Department of Radiology, University of California, San Diego Medical Center, San Diego, CA USA; 3 Department of Radiology, Hospital of the University of Pennsylvania, Philadelphia, PA USA; 4 Department of Radiology, Penn State Hershey Medical Center, Hummelstown, PA USA; 5 Department of Radiology, New York University Grossman School of Medicine, New York, NY USA; 6 Summit Physician Specialists, Murray, UT USA; 7 GNMI in Mississauga, Greater Toronto Area, Toronto, ON Canada; 8 Department of Radiology, Division of Musculoskeletal Imaging, Olympia Medical Center, Elite Sports Imaging, Madrid, Spain; 9 Department of Radiology, University of California, Los Angeles Medical Center, Los Angeles, CA USA; 10 Department of Radiology, Mayo Clinic, Rochester, MN USA; 11 Department of Radiology, University of Colorado School of Medicine, Aurora, CO USA; 12 Department of Radiology, Mayo Clinic, Rochester, MN USA; 13 McCaig Institute for Bone and Joint Health, Calgary, Canada; 14 Cumming School of Medicine, University of Calgary, 3280 Hospital Dr NW, Calgary, AB T2N 4Z6 Canada

**Correction to: Skeletal Radiology**

10.1007/s00256-023-04420-6

The correct name should be Michael D. Ringler.

The correct affiliation of Dr French is

Department of Radiology, Penn State

Hershey Medical Center, Hummelstown, PA

The original article has been corrected.

